# Effects of whole-body vibration on postural control in elderly: a systematic review and meta-analysis

**DOI:** 10.1186/1471-2318-11-72

**Published:** 2011-11-03

**Authors:** Slavko Rogan, Roger Hilfiker, Kaspar Herren, Lorenz Radlinger, Eling D de Bruin

**Affiliations:** 1 Bern University of Applied Sciences - Health, Bern, Switzerland; 2 University of Applied Sciences Western Switzerland, Leukerbad, Switzerland; 3 Bern University Hospital, Bern, Switzerland; 4 Institute of Human Movement Sciences and Sport, ETH Zurich, Zurich, Switzerland

## Abstract

**Background:**

This systematic review was performed to summarize the current evidence for whole body vibration (WBV) interventions on postural control in elderly.

**Methods:**

English and German language papers in Medline, PEDro, Cinahl and the Cochrane databases were searched. Two reviewers extracted data on patients' characteristics, type of WBV intervention and outcomes. Two independent reviewers rated the methodological quality of these studies. Data were pooled using random-effects meta-analysis.

**Results:**

Fifteen papers reporting quantitative data were included. Results from 15 papers could be pooled for a meta-analysis. The studies involved 933 participants. In 7 studies the authors investigated the effects of vibration plates generating vertical sinusoidal vibrations (VS-WBV) and 7 papers described the use of side-alternating sinusoidal vibrations (SS-WBV). One study investigated both VS-WBV and SS-WBV.

Weak to moderate evidence of an overall effect as a result of VS-WBV and SS-WBV was observed for (a) static balance for post-intervention values with a standardized mean difference (SMD) -0.06, 95% CI -0.31 to 0.18 and for change values SMD -0.26, 95% CI -1.09 to 0.57, and (b) dynamic balance for post-intervention-values SMD -0.34, 95% CI -0.60 to -0.08. For functional balance (c) an overall outcome for post-intervention values with SMD of 0.34, 95% CI -0.19 to 0.87 was found.

**Conclusions:**

The 15 studies reviewed were of moderate methodological quality. In summary, SS-WBV seems to have a beneficial effect on dynamic balance in elderly individuals. However, the current results should be interpreted with caution because of the observed heterogeneity of training parameters and statistical methods. Future studies are warranted to evaluate the effects of WBV on postural control in an elderly population.

## Background

Even in the absence of overt pathology, motor functioning [cf. International Classification of Functioning (ICF) (see http://www.who.int/classification/icf)] can deteriorate, as is illustrated by more frequent falls in ageing populations [1]. Usually multi-factorial disorders such as impaired vision, vestibular dysfunction, sensory loss, muscular weakness or gait disorders contribute to more frequent falls [2-5]. Falls are amongst the most common reasons for medical intervention in the elderly and their occurrence may initiate a vicious circle that causes fear of falling, nursing home admittance and loss of independence [6]. About 30% of the elderly fall at least once a year, while one-fifth of these need medical care [7].

Because a significant portion of the older population is unable or unwilling to comply with conventional training regimens [8], there seems to be a need for a search and assessment of alternative forms of training intervention contents. More recently, whole body vibration (WBV) training has been widely used in fitness centers, sports, and physical therapy to improve cardiorespiratory fitness, power and strength [9-16] or bone mineral density [17-21]. Several studies have demonstrated that WBV also improves postural control in healthy young or elderly individuals as well as in patients with orthopedic (e.g. rupture of the cruciate ligaments) [15] or neurological diseases (e.g. Parkinson`s disease, multiple sclerosis, spinal cord injury) [14,22,23]. Torvinen et al. [24] showed beneficial long-term effects of sinusoidal WBV on the strength of young healthy individuals but not immediately on postural control. For patients with Parkinson`s disease Haas and Schmidtbleicher postulated [25] that stochastic resonance whole-body vibration (SR-WBV) transiently improves balance.

However, while most WBV studies demonstrate significant improvements in balance, these results must be interpreted with caution. Many of the published studies have methodological flaws such as the questionable validity of the outcome measures used or the absence of a sham intervention [26]. The results of the studies are not completely consistent, and some reports found only little or no effect on postural control following WBV training [18,24,27,28]. One explanation for the contradictory results could be attributed to the inconsistent training parameters used for WBV training. The frequency [11], amplitude, duration of one vibration session, and the number of vibration interventions, are the treatment parameters that need to be considered when using WBV. The duration of rest periods between vibration sessions also seems to play an important role [29,30]. It is, furthermore, very difficult to determine an optimal training strategy because the underlying mechanisms contributing to improved balance after WBV have so far not been clarified. Most of the devices used vibrate sinusoidally while one system generates stochastic vibrations. The devices which induce sinusoidal vibrations have subjects standing on one platform and they either oscillate purely vertically (VS-WBV) or side-alternating (SS-WBV). The WBV device that vibrates in a stochastic manner (SR-WBV) exhibits separate platforms for each foot.

The purpose of this systematic review is to provide an overview of the current available evidence for the use of WBV to improve balance in elderly individuals. In particular the following aspects should be clarified: a) assessment of the quality and internal validity of the included studies, b) description of the assessments used to document the effects of WBV on balance, c) composition of the WBV training parameters in relation to the different vibration plates, and d) conclusion about the clinical relevance. Furthermore this review should give some more conclusive results about the effects of WBV on the balance skills of elderly by summarising the available studies in a meta-analysis.

## Methods

### Data Sources and Searches

The methods of the analysis and the inclusion criteria were developed and documented in a protocol prior to the actual review. This protocol can be found in additional file 1. PRISMA guidelines were followed for this systematic review and meta-analysis [31]. An electronic search of the following databases up until May 2011 was conducted: PubMed, Cochrane Register of Controlled Trials, Physiotherapy Evidence Database (PEDro) and CINAHL (Ebsco Host). The unpublished International Clinical Trials Registry Platform from the World Health Organization (WHO) was also searched. Furthermore a manual search was completed within the reference lists of retrieved publications.

### Study Selection and Research Question

This systematic review was conducted to answer the question formulated according recommendations from the PICO-model, where the acronym PICO stands for Population (in the actual review: elderly), Intervention (WBV exercise; WBV training parameters), Comparator (no or other balance enhancing exercise) and Outcomes (examination of postural control; static, dynamic, functional and balance; falls) [32].

The following keywords were used for formulating the search strategy of our review:

Population: elderly, aged, dwelling home, nursing home

Intervention: Whole-body vibration, WBV, noise, random vibration

Outcome: Balance, postural stability, postural control, sway, falls

In the case of missing data additional information was requested from the corresponding authors of relevant papers in order to include these data in our meta-analysis.

Three independent reviewers (SR, KH, RH) screened the titles and abstracts for eligibility. We were aiming at Randomized Controlled Trials (RCT) measuring postural control/balance in studies using WBV intervention in elderly subjects. Both published and unpublished (grey literature) full text articles in English or German were eligible for inclusion. Elderly participants and all clinical outcome measures of static, dynamic and functional balance performance as well as computerized biomechanical assessments of postural control (e.g. posturography) were included in this review. Detailed descriptions of the different balance tests are reported elsewhere [33-36]. The following types of articles were excluded: studies describing vibrations applied by current or vibrating insoles and conference papers.

### Data Extraction and Quality Assessment

The methodological quality of the included articles was rated with "The Cochrane Collaboration's tool for assessing risk of bias". The criteria list comprised six items. Each item was scored with "+" if the criterion was fulfilled, with "-" if the criterion was not fulfilled, and with "?" if the information was not provided or was unclear. All included papers were scored independently by three reviewers (KH, RH, SR). Discrepancies were resolved by discussion and consensus.

In addition, general characteristics of the studies were extracted. Two authors (KH, SR) independently abstracted the following information from each of the studies included in this review: 1) design and sample; 2) inclusion criteria; 3) training parameters 4) type of vibration plate; 5) change in static, dynamic and functional balance and 6) conclusions of the studies and statistical significance.

### Data Synthesis and Analysis

All outcomes of interest were presented as continuous data (mean values and SD or mean changes). We used standardized mean difference (SMD), except for the analysis of the Timed up and Go test (TUG) where we used weighted mean difference (WMD). Random effects models were applied. The magnitude of the effect size SMD can be rated as follows: 0.2 indicates a small effect, 0.5 a moderate effect and 0.8 a large effect [37]. Where only one study was identified or data were not eligible to be included in the meta-analysis, results of individual studies are presented.

To determine the isolated effect of WBV on posture we additionally performed a sensitive analysis for dynamic balance where studies with WBV in combination with exercise were compared to studies with isolated WBV interventions.

If studies reported more than one balance assessment, the primary outcome of a study was chosen and included in the meta-analysis.

Heterogeneity was assessed by forest plots and the I^2 ^statistics. Values > 25% indicate small, > 50% middle and > 75% considerable heterogeneity [38]. All other information was summarized and analyzed qualitatively.

For all calculations Stata (Version12) was used.

## Results

### Study characteristics

The literature search revealed a total of 95 possibly eligible papers (PubMed: n = 88, Cochrane: n = 30, CINAHL: n = 12, PEDro: n = 13, and Unpublished International Clinical Trials Registry Platform: n = 33). The title and abstracts of these 95 papers were studied and after removing duplicates 71 studies remained for further analysis. The majority of these papers had to be excluded (n = 56) because they did not refer to postural control or used electrical vibration stimuli. Finally 15 full text papers could be included in the present meta-analysis [18,21,39-51].

7 papers reported vertical sinusoidal WBV (VS-WBV) and 7 papers side-alternating sinusoidal WBV (SS-WBV). One study reported on a combined VS-WBV and SS-WBV intervention [51]. For SR-WBV, no RCT studies could be identified.

**Figure 1:**
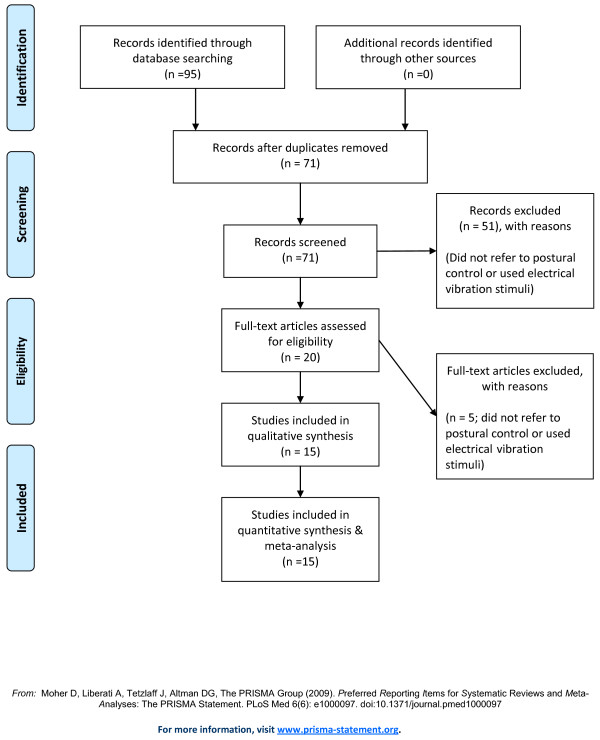
**Results of the systematic review**. Studies' flow chart for the review and meta-analysis.

A flow diagram of the search process is shown in Figure 1.

### Study Design and Characteristics of Population

A broad quality spectrum within the RCT study design and heterogeneity within the applied training strategies was found. The major characteristics of the 15 selected papers are summarized in Table 1.

**Table 1:** Overview of Selected Whole-Body Vibration studies on postural control

Study	Subjects	Study Design	Mean age	N: gender	Duration of WBV training	Parameters WBV	Parameters Control (CON)	Device	Outcome Measures	Main Results
**Sinusoidal Vertical Vibrations**										
Bautmans et al. 2005 [40]	Nursing home residents	RCT	WBV:76.6 CON:78.6	N = 24: 15♀/9♂	6 w	FRQ: 4 × 30-60 s WBV/session 30-60 s rest in between 3 sessions/w F: 30-40 Hz A: 2/5 mm POS: Static exercises while WBV	Static exercises without WBV 3 sessions/w	Power Plate	TUG Tinetti Test	WBV/CON: Significant difference in the improvement between WBV and CON for both balance tests (TUG & Tinetti).
Beck et al. 2010 [51]	Postmenopausal women	RCT	WBV:68.9 CON:74.2	N = 47♀	8 m	FRQ: 15 min LWBV/1 session no rest 2 sessions/w F: LWBV: 30 Hz A: 0.3 g POS: standing with full extension	No vibration	Juvent	SLS Tandem Walk Test	WBV/CON No improvements of some aspects of postural control
Bogaerts et al. 2007[42]	Community-dwelling elderly	RCT	WBV: 66.9 FIT: 67.6 CON: 68.6	N = 220: 106♀/114♂	52 w	FRQ: 4 × 30 s-15 × 60 s WBV/session 15-60 s rest in between 3 sessions/w F: 30-40 Hz A: 2.5/5 mm POS: Static/dynamic exercises while WBV (squats, toe-stand etc.)	FIT: 1.5 h cardiovascular, strength, balance, flexibility exercises (running, cycling, strength etc.) 3 sessions/w CON: No change in lifestyle	Power Plate	Sensory Organization Test (SOT)	WBV: Improvement of some aspects of postural control
Boegarts et al. 2011 [41]	Elderly women	RCT	WBV:80.3, 79.8 CT: 78.7, 79.6	N:113 113♀	6 m	FRQ: 3 × 15 s - 60 s with 60 to 5 s rest between per week F: start 30 and end 40 Hz A: 1.6 - 2.2 g POS: exercises squat, deep squat, wide stance squat, toes stand, one leg stance	Control: no change in lifestyle	Powerplaate	Static balance on forc, dynamic balance (SOT), TUG,	WBV: Sway velocity decrease significantly with open and closed eyes in static balance. No changes in dynamic balance. TUG significantly decrease in both groups
Carlucci et al. 2010 [39]	Elderly women	Quasi RCT	WBV: 71.8 CON: 71.4	N = 36♀	One session	FRQ: 6 min 3, 5 min rest F: 35 Hz POS: Static and dynamic knee-extensor exercises.	Static and dynamic knee-extensor exercises without vibration.	Well-net Vibe Revolution	Posturography	WBV: No significant improvement in static balance after WBV.
Johnson et al. 2010 [43]	Patients after total knee arthroplasty [57]	Quasi RCT	WBV:67 CON:68.5	N = 16 6♀/10♂	4 w	FRQ: began at 2 min (1 × 30 s) and progressed to 18 min (6 exercises, 3 × 30 s) 3 sessions/4 F: 35 Hz A: 2 mm (1 + 2 w) 2- 5 mm (3 w) 5 mm (4 w) POS: strengthening exercises on a WBV platform	Traditional progressive resistive exercise	Power Plate	TUG	WBV: Significant improvement was 31% TPRE: Significant improvement was 32%
Mikhael et al. 2010 [44]	elderly	RCT	WBV FK: 63.3 WBV LK: 69 Sham: 62.3	N = 19 11♀/8♂	13 w	Group WBV with flexed knees [58] and Group WBV with locked knees (LK) FRQ: 10 × 60 s WBV/session 60 s rest between 3 sessions/w A: 12 Hz F: 1 mm (peak to peak) POS: Stood on the platform with feed shoulder-width apart, hands by their side	Sham: with flexed knees A: 12 Hz F: 1 mm		Balance measured by balance index, was assessed on a force platform	WBV/Sham: No improvement of the balance index
Verschueren et al. 2004 [18]	Postmenopausal women	RCT	WBV: 64.6 RES: 63.9 CON: 64.2	N = 70: 70♀	24 w	FRQ: WBV overload principle: Varying number/durations of vibration bouts and rests ≤ 30 min/session 3 sessions/w F: 35-40 Hz A: 1.7/2.5 mm POS: Static/dynamic knee-extensor exercises while WBV (Squats, lunge etc.)	RES: Knee-extensor exercises on leg-extension and leg-press according to overload principle: 60 min/session 3 sessions/w CON: Maintain actual level of physical activity, no training	Power Plate	Bertec^® ^force plate measuring body sway under static and dynamic (arm abduction or flexion while standing) conditions	WBV: Significant reduced body sway under dynamic conditions after WBV (p < 0.05). Between group difference for change over time only for the dynamic conditions compared to CON (p = 0.003/p = 0.03). CON: No change over time
**Side-alternating Vibration**										
Beck et al. 2010 [51]	Postmeno- pausal women	RCT	HWBV:68.5 CON:74.2	N = 47♀	8 m	FRQ: 2 × 3 min HWBV/session 60 s rest in between 2 sessions/w F:12.5 Hz A: ~ 2 mm POS: standing with slightly bended knee	No vibration	Galileo	SLS Tandem Walk Test	WBV/CON No improvements of some aspects of postural control
Bruyere et al, 2005 [45]	Nursing home residents	RCT	WBV: 84.5 CON: 78.9	N = 42: 31♀/11♂	6 w	FRQ: 4 × 60 s/session 3 sessions/w 90 s rest in between F: 10/26 Hz A: 3/7 mm POS: Static standing while WBV + Additional physical therapy: (gait, balance, ADL, strength)	Physical therapy (gait, balance, ADL, strength) 3 sessions/w	Galileo	TUG Tinetti Test: Balance score	WBV: Significant greater improvement in both balance tests compared to CON.
Cheung et al. 2007 [46]	Elderly healthy women	RCT	WBV: 72.5 CON: 72.0	N = 69♀	12 w	FRQ: 3 min/session 3 sessions/w F: 20 Hz A: 0-5.3 mm (model specifications) POS: Static standing while WBV	Remain sedentary Normal daily life throughout the whole study	Galileo	Basic Balance Master system: Limits of stability of COP (Movement velocity/maximum excursion/directional control) Functional Reach Test	WBV: Significant difference in change compared to CON on Basic Balance Master system Tendency to greater improvement compared to CON in Functional Reach
Furness et al. 2009 [47]	Elederly, community-dwelling adults	RCT	WBV: 72 ± 8	N = 73 38♀/35♂	6 w	FRQ: 5 × 60 s WBV/session 60 s rest in between Group A: 1 session/w Group B: 2 sessions/w Group C: 3 sessions/w F: 15-25 Hz A: 0.5 mm POS: Static standing while WBV with holding on handlebars (110° knee extension)	No WBV		Tinetti-Test TUG	Group B+C: Significant improvement TUG and Tinetti Test. Group C significantly greater improvements for the TUG and Tinetti Test than group B.
Furness et al. 2010 [48]	Elederly, community-dwelling adults	RCT	69 ± 8	N = 37 21♀/16♂	6 w	FRQ: 5 × 60 s WBV/session 60 s rest in between F: 15 Hz for first 6 session F: 20 Hz for 6 session F: 25 HZ for last 6 session A: 1 mm POS: Static standing while WBV with holding on handlebars (70° knee flexion)	No WBV and no additional form of exercise		TUG	WBV elicited beneficial adaptions in functional performance
Gusi et al. 2006 [21]	Postmenopausal women, untrained	RCT	WBV: 66 CON: 66	N = 28♀	32 w	FRQ: 3-6 × 60 s WBV/session 60 s rest in between 3 sessions/w F: 12,6 Hz A: 3 mm POS: Static standing while WBV (60° knee flexion)	55 min walking + 5 min stretching	Galileo	Blind Flamingo Test	WBV Improved balance (29%) CONt Balance did not improve
Rees et al. 2007 [49]	Healthy elderly persons	RCT	WBV: 74.3 EX: 73.1 CON: 73.1	N = 43: 20♀/23♂	8 w	FRQ: 6 × 45-80 s WBV/session 45-80 s rest in between 3 sessions/w F: 26 Hz A: 5-8 mm POS: Static/dynamic exercises while WBV (squats, calf raises etc.) + ≥ 3x/w low intensity exercise (walking)	EX: Static and dynamic exercises (squats, calf raises etc.) without WBV 3 sessions/w + ≥ 3x/w low intensity exercise (walking) CON: ≥ 3x/w low intensity exercise (walking)	Galileo	Timed-Up-and Go (TUG) Sit-to-Stand (STS)	WBV: Significant difference in amount of change in TUG compared to CON WBV/EX: Significant difference in amount of change in STS compared to CON
Rees et al.2009 [50]	Healthy elderly persons	RCT	WBV: 74.3 EX: 73.1 CON: 73.1	N = 43: 21♀/24♂	8 w	FRQ: 6 × 45-80 s WBV/session 45-80 s rest in between 3 sessions/w F: 26 Hz A: 5-8 mm POS: Static/dynamic exercises while WBV (squats, calf raises etc.) + ≥ 3x/w low intensity exercise (walking)	EX: Static and dynamic exercises (squats, calf raises etc.) without WBV 3 sessions/w + ≥ 3x/w low intensity exercise (walking) CON: ≥ 3x/w low intensity exercise (walking)	Galileo	One-legged postural steadiness (OLPS) Timed-Up-and Go (TUG)	WBV: revealed significant improvements for the VIB group compared to the EX and CON groups

Abbreviations: N: Number/RCT: Randomized controlled trial/WBV: Whole body vibration/CON: Control group/RES: Resistance training group/EX: Exercise group/FRQ: Frequency of training/F: Frequency of vibration platform/Hz: Hertz/A: Amplitude/POS: Initial position/s: seconds/min: minutes/d: day/w: week/?: No description in study

Studies were conducted in Australia (6), Belgium (5), Italy (1), Hong Kong (1), Spain (1), and the USA (1).

### Methodological Quality

All included studies summarized in table 2 showed a bias following the "Cochrane Collaboration's tool for assessing risk of bias". Most of them had a very high risk for "allocation concealment", "blinding", and for "incomplete outcome data". Only four of the included studies [41,44,46,48] provided some information about randomization procedures, suggesting that randomization was probably concealed. Two studies [40,44] report a blind assessor incorporated for all outcome measures. Most studies had a low risk for "incomplete data" and "other bias".

**Table 2:** Methodological quality of included trials

Study	RCT	Allocation Concealed	Blinding	Incomplete data addressed	Free of selective reporting	Free of other bias
Bautmans et al. 2005 [40]	+	-	+	+	+	+
Beck et al. 2010 [51]	+	.	.	+	+	?
Bogaerts et al. 2007 [42]	+	-	-	-	+	+
Boegarts et al. 2011[41]	+	+	-	+	+	+
Carlucci et al. 2010 [39]	quasi RCT	-	-	+	+	+
Johnson et al. 2010[43]	quasi RCT	-	-	+	+	+
Mikhael et al. 2010 [44]	+	+	+	+	+	+
Verschueren et al. 2004 [18]	+	-	?	+	+	+
Bruyere et al. 2005 [45]	+	-	-	+	+	+
Cheung et al. 2007 [46]	+	+	-	-	+	-
Furness et al. 2009 [47]	+	?	-	+	+	-
Furness et al. 2010 [48]	+	+	-	+	+	?
Gusi et al.2006 [21]	+	-	-	-	+	+
Rees et al. 2007 [49]	+	-	-	-	+	+
Rees et al. 2009 [50]	+	-	-	-	+	+

Abbreviations: +: criterion was fulfilled/-: the criterion was not fulfilled/?: the information was not provided or was unclear

### Training Protocols

Table 1 shows WBV training protocols of the included studies (Table 1). Several differences were seen in the WBV training protocols. Treatment parameters for VS-WBV revealed frequencies ranging between 12 to 40 Hz. The most common mean frequency was about 30 Hz. A training session usually consisted of 3 to 10 series of 30-60 seconds WBV with a rest of 30 to 60 seconds in between. Beck et al. [51] described one single session lasting 15 minutes without rest. For long-term interventions the authors often prescribed 3 WBV sessions per week. Verschueren et al. [18] used variable numbers of sessions per week. The duration of long-term WBV lasted from 6 to 52 weeks. The initial position the subjects had to adopt during WBV differed between the studies. One author prescribed standing upright statically with feet shoulder-width apart and hands at the side [44]. In a further six studies [39-43,51,52] the participants had to perform various dynamic physical exercises during WBV (e.g. squatting exercises).

Treatment parameters for SS-WBV revealed frequencies ranging between 5 and 26 Hz. A training session usually consisted of 3 to 10 series of 30-60 seconds WBV with 60 seconds rest in between. Cheung et al. [46] did not describe the vibration session in detail (time of intervention/rest). Bruyere et al. [45] used 90 seconds, and Rees [49] used 45 to 80 seconds rest time between the vibration bouts. Frequency of training was 3 sessions per week for long-term intervention. Furness et al. [47] described three different vibration groups which trained 1, 2 or 3 times per week. The duration of the whole WBV intervention varied between 6 to 32 weeks. Comparable with the VS-WBV studies, the starting positions of the subjects on the vibration devices varied. In most of the studies the participants stood in an upright position with slightly bent knees. Two studies prescribed dynamic exercises during WBV [49,50].

### Outcome Measures

In total, 10 different outcome measures for postural control/balance could be found in the 15 studies. For VS-WBV, different assessments for static balance (Posturography on force plates, Balance index, single leg stand), for dynamic balance (Timed Up and Go (TUG) Test, Sensory Organization Test (SOT), Tandem walk test) and for functional balance (Tinetti Test/POMA) could be found.

One RCT reported no improvement in static and dynamic balance after WBV. In this study subjects were not expected to perform active exercises whilst standing on the vibration plate. Two RCTs [18,41] observed improved static balance after WBV combined with simultaneous dynamic exercises. One RCT [44] reported no improvement after vibration without exercise in the balance index. Three RCTs [40-42] described improvement in dynamic balance following a combination of vibration with exercise. One RCT [40] reported improvement in functional balance following vibration bouts that were combined with exercise.

For the studies that applied SS-WBV several outcome measures were used. Three assessments for static balance (Basic Balance Master System, Blind Flamingo, single leg stand), 4 for dynamic balance (Functional Reach Test [FRT], Timed Up and Go [TUG] Test, chair rising, tandem walk test) and 1 for functional balance (POMA) were described.

Two RCTs [21,46] reported improvements in static balance of the WBV participants. These improvements were achieved without performing additional dynamic exercises during WBV. Five RCTs [45-49] showed improved balance in subjects of the WBV group which was not obligated to perform simultaneous active exercises. Two RCTs [49,50] demonstrate improvements in dynamic balance after a combination of WBV with additional exercises on the vibration platform. Two RCTs [45,47] report improvements in functional balance after WBV without additional exercises. One study found no improvements in neither static nor dynamic balance after isolated WBV [51].

### Meta-analysis

**Figure 2:**
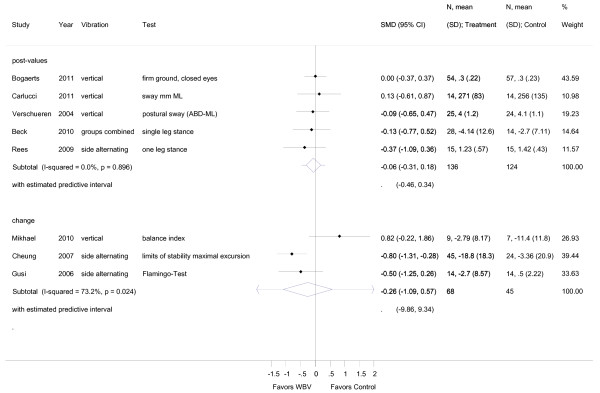
**Forest plot of 8 trials comparing the effects of any type of vibration and control interventions on static balance**. The analyses were separated for trials reporting post-values (i.e. mean and SD from follow-up) and for trials that reported change values (i.e. mean and SD from the changes from baseline to follow-up). Random effects model with predictive interval. The predictive interval indicates the range within which we expect the effects of 95% of future studies. Values on x-axis denote SMDs.

**Figure 3:**
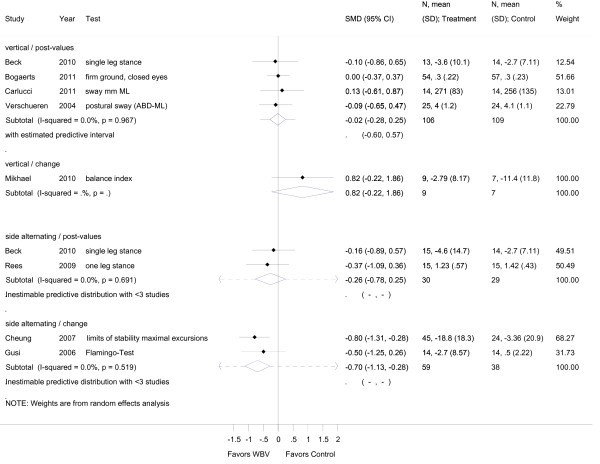
**Forest plot of 8 trials (9 comparisons) stratified for the vibration type (vertical and side alternating)**. Outcomes were tests for static balance. The analyses were separated for trials reporting post-values (i.e. mean and SD from follow-up) and for trials that reported change values (i.e. mean and SD from the changes from baseline to follow-up). Random effects model with predictive interval. The predictive interval indicates the range within which we expect the effects of 95% of future studies. Values on x-axis denote SMDs.

**Figure 4:**
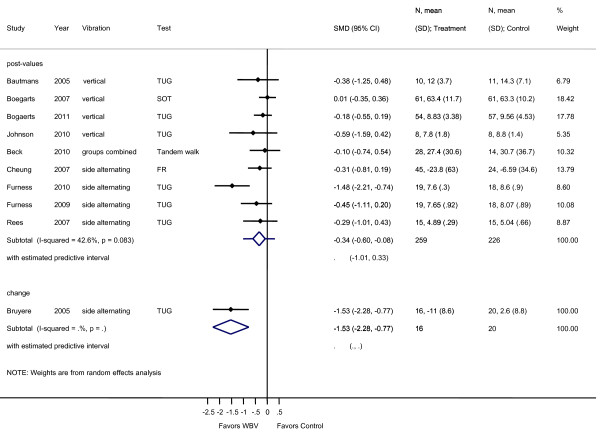
**Forest plot of 9 trials comparing the effects of any type of vibration and control interventions on dynamic balance**. The analyses were separated for trials reporting post-values (i.e. mean and SD from follow-up) and for trials that reported change values (i.e. mean and SD from the changes from baseline to follow-up). Random effects model with predictive interval. The predictive interval indicates the range within which we expect the effects of 95% of future studies. Values on x-axis denote SMDs.

**Figure 5:**
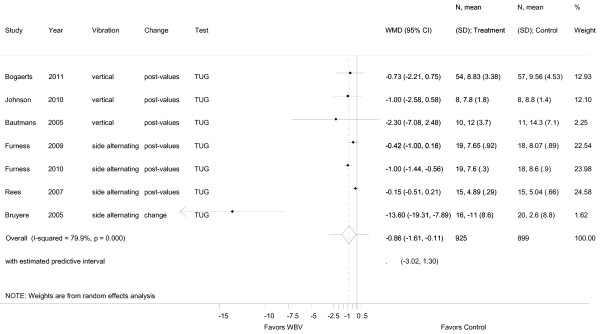
**Forest plot of 7 trials comparing the effects of any type of vibration and control interventions on the Timed Up and Go Test**. Random effects model with predictive interval. The predictive interval indicates the range within which we expect the effects of 95% of future studies. Values on x-axis denote WMD (weighted mean difference).

**Figure 6:**
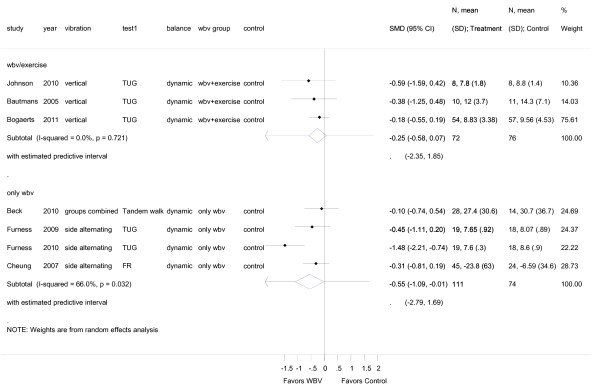
**Forest plot of 6 trials comparing overall effects of WBV-plus-exercise versus isolated WBV**. The analysis reports post intervention values. Values on x-axis denote SMDs.

For the meta-analyses, 15 studies were included which described static, dynamic or functional balance outcome measurements. The effect sizes for these outcomes are summarized in Figures 2, 3, 4, 5 and 6.

### Static balance

The mean overall effect size for post-intervention values of static balance was SMD -0.06 (95% CI -0.31 to 0.18) and for change values SMD -0.26, 95% CI -1.09 to 0.57 (SS-WBV) (Figure 2). Post-intervention values for dynamic balance was SMD -0.34 (95% CI -0.60 to -0.08) (Figure 4), and for functional balance an overall outcome for post-intervention values revealed a SMD of 0.34, 95% CI -0.19 to 0.87 (additional file 2). There was small heterogeneity for post-intervention values for static balance with I^2 ^0.00% (*p *= 0.896), middle heterogeneity with I^2 ^73.2% (*p *= 0.024) for change values, and small heterogeneity for post-interventional values in dynamic balance with I^2 ^42.6% (*p *= 0.083). For functional balance no heterogeneity with (I^2 ^3.4%, *p *= 0.309) was found.

For subgroup analysis, variables were pooled separately for VS-WBV and SS-WBV. Four VS-WBV studies showed post intervention mean values indicating a small effect size (SMD -0.02, 95% CI -0.28 to 0.25) for static balance (Figure 3). There was no significant heterogeneity between these studies (I^2 ^0.0%, *p *= 0.97). Two studies investigating SS-WBV reported small effect sizes for static balance (SMD -0.26, 95% CI -0.78 to 0.25), with no significant heterogeneity (I^2 ^0.0%, *p *= 0.691). For change values two studies reported moderate effect sizes (SMD -0.70, 95% CI -1.13 to 0.28) and no significant heterogeneity (I^2 ^0.0%, *p *= 0.519).

### Dynamic balance

For dynamic balance outcomes and VS-WBV post intervention mean values (additional file 3) five studies reported a small effect size (SMD -0.014, 95% CI -0.3 to 0.17) favoring vibration training. No significant heterogeneity was found (I^2 ^0.0%, *p *= 0.48). In five papers reporting SS-WBV, a small to moderate effect size (SMD -0.49, 95% CI -0.94 to -0.05) in favor of vibration training and evidence for middle heterogeneity (I^2 ^56.6%; *p *= 0.056) was found.

Pooling of VS-WBV with SS-WBV in all studies reporting on TUG (n = 7) resulted in a weighted mean difference (WMD) of -0.86, (95% CI -1.61, -0.11). Considerable heterogeneity (I^2 ^79.9%; *p *= 0.000) was shown in this case (Figure 5).

**Figure 7:**
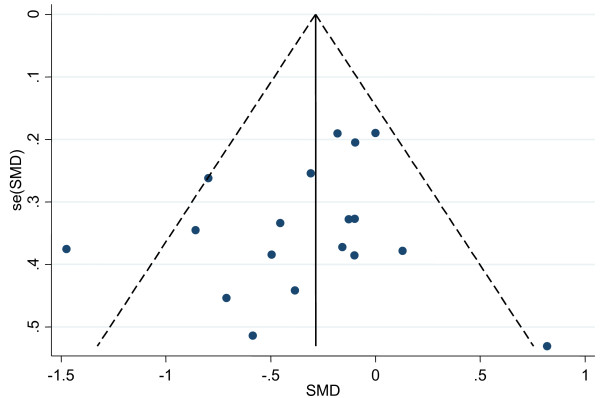
**Funnel plot of included WBV trials**. A funnel plot with all points evenly distributed on both sides of the solid vertical line indicates no publication bias.

In an attempt to discriminate between WBV-plus-exercise-trials and WBV-without-exercise-trials we performed a subgroup analysis comparing overall effect sizes from studies reporting on dynamic balance with WBV combined with exercise versus WBV only (Figure 6). WBV-plus-exercise revealed a small effect size of SMD -0.25 (95% CI -0.58 to 0.07) in dynamic balance. WBV-without-exercise revealed a moderate effect size of SMD -0.55 (95% CI -1.09 to -0.01). We also plotted a funnel plot (Figure 7) and performed an egger's test for small study bias (i.e. including the potential of publication bias). There was no evidence for small study bias neither in the funnel plot nor in the egger's test (*p *= 0.367).

## Discussion

This systematic review of 15 studies included a total of 933 participants. The main aims were to estimate the quality and internal validity of the studies and to describe the balance assessments used to assess the effect of WBV training. Furthermore we aimed to provide some information about the clinical relevance of the studies. Following the results of the meta-analysis, it can be postulated that a side-alternating WBV (SS-WBV) intervention can improve dynamic balance in elderly subjects. This is based on the position of the diamond lying left of the 'no difference' line that indicates that WBV is beneficial. This finding is despite much variation in protocol design of the included studies and is, thus, encouraging that SS-WBV may be a viable precursor to more traditional forms of exercise training aiming to improving balance and function among sedentary and frail older adults. However, training using VS-WBV revealed only small effects for static (a) and dynamic balance (b), while SS-WBV showed small to moderate improvements for the same balance requirements. These findings seem to suggest that the vibration pattern of WBV platforms has differing influence on the postural outcomes. These differing findings might, however, also be a result of the still rather small amount of WBV studies available that were performed in elderly populations. This systematic review, therefore, only reveals first estimates for these measures and warrants further WBV research in larger populations. This in mind, the relationship between research with different WBV systems and their effect(s) on postural control in elderly individuals requires further exploration. Translating the results from WBV experiments with healthy elderly participants to therapeutic interventions should, therefore, take place with caution until the appropriate clinical studies with clinically relevant population outcomes have been conducted. For this reason no clear recommendations for clinical use of WBV to improve dynamic balance in elderly can at present be made. These findings rather warrant further research in to the effects of SS-WBV on balance of elderly with sufficiently powered RCT study designs.

During WBV training that is combined with performance of active exercises theoretically two different stimuli for the muscle-nerve system can be identified and made responsible for the observed training effects: [I] reflex muscle contraction induced during vibration and [II] body weight exercises. This makes it difficult to attribute observed effects to the WBV training in those studies that use a combined training approach. However, the recently published meta-analysis of Steib et al. [53] showed that more traditional forms of resistance training have no effects on postural control. Our analysis where we separate the studies with WBV-plus-exercise-trials from WBV-without-exercise-trials shows that also SS-WBV without exercise shows a similar direction and magnitude of effect sizes and, therefore, indicates to have a positive influence on dynamic balance.

The frequency (F), amplitude (A), frequencies of training sessions per week (FRQ) of vibration stimuli either applied with SV-WBV or SS-WBV and their respective effect on balance outcomes reveals varying results. Low frequencies from 10 to 26 Hz showed higher effectiveness than frequencies between 30 to 40 Hz. Most applications with frequencies between 10 to 30 Hz were performed on SS-WBV devices. The amplitudes vary from 0.5 mm to 8 mm in both types of vibration. The amplitudes seem not to have a significant role. Frequencies over 30 Hz generate a faster platform movement and deliver more energy to the body.

FRQ describes the total time on the WBV device per duration of the vibration sessions, the total rest time between the duration of the vibration sessions and the sessions per week. The optimal time per workout with SS-WBV for static and dynamic balance appears to be: 3 vibration sessions of 60 seconds with 60 seconds rest time between each and a frequency of 3 times per week. For VS-WBV it is unclear which FRQ has to be used in order to attain optimal training results, because the different studies were applying different training parameters. For instance, it is unclear whether shorter sessions (3 sets of 15 seconds) or longer sessions (15 sets of 60 seconds) or sessions performed without a break lasting 15 minutes [51] are most effective. Future research must examine these aspects for VS-WBV. In addition it is unclear whether loading parameters of 0.3 g (low intensity) performed with VS-WBV are comparable in effect to loading parameters of 0.45 g to 0.8 g with SS-WBV which seems to have no effect on balance. However, during SS-WBV the loading parameter started with low-intensity and was increased to high-intensity WBV during the course of the training period, thus adding a form of progression to the training [47,48]. This is another aspect that should be focused on in future studies.

A point of attention in future studies should be the inclusion of both men and women in WBV studies when postural control is of concern. Four studies with VS-WBV [18,39,41,51] were conducted with women only. For SS-WBV two studies [21,46] with women only were conducted. All other studies were conducted with women and men. This makes it difficult to generalize the findings of this review to both genders due to the relative low amount of studies with information on men. Another point of concern is related to the age of the participants. Only three studies include a sample of volunteers over the age of 75 years. The age of 75 years, however, seems to be a threshold point for problems with postural control. Especially over 75 years of age health-related causes gain greater importance as causes for falls. The fact that this age group was not studied in the majority of the included studies might hypothetically explain the lack of observed effects in some of the studies.

### Study limitations

We developed and utilized a structured study protocol to guide our search strategy, study selection, extraction of data and statistical analysis. However, a number of possible limitations of this review should be noted. First, a publication bias may be present, as well as a language bias, given that we restricted our search to English and German language publications. Second, we included 15 randomized trials. The risk of bias for these trials shows a high risk of bias for allocation concealment and blinding. Finally, the interventions in these trials were of relatively short duration when we consider the time of training in individual sessions and heterogeneous in their design. A Cochrane review that investigated exercise for improving balance in older people [35] showed that in general successful programs offer exercise sessions from 15-20 minutes up to 70 minutes per session that are performed over periods spanning from 5 weeks up to 12 months [35]. Compared to this many WBV studies offer exercise sessions that are rather short in duration.

Another item that should be critically viewed is the underlying assumption that postural balance measures are related to future falls in elderly populations. Falling is a complex phenomenon and, among elderly people, both intrinsic and extrinsic risk factors must be evaluated. Poor balance is assumed to be one of the major risk factors for falls among the elderly. According to the results of a recent review there indeed seems to be a relation between force platform parameters (derived in laboratory circumstances) and future falls. However, this relationship has mainly been derived from cross-sectional study designs, and we cannot state that this is also a causal relation as implied in this systematic review. The number of prospective studies relating measures of postural control with falls is in fact relatively low and the variation in the ways the various postural control assessments; i.e., force platform measurements [54], have been carried out and how the results have been analyzed in each case makes it difficult to draw definitive conclusions.

### Future research

Despite these limitations, we believe that our review provides useful information regarding the effects of WBV interventions aimed at improving postural control in the elderly. It also provides some guidance about the components that should be explicitly considered in future interventions to enhance their effect on postural control. Future studies evaluating the effects of WBV interventions should preferably involve RCT type studies carried out among diverse sub-populations of the elderly. Primary outcomes for such RCT's should, as previously suggested, include both physical activity and detailed evaluation of postural control related health outcomes assessed both in the short and longer term. Trials that ultimately intend to reduce the number of falls in their study population should preferably adopt the Prevention of Falls Network Europe (ProFaNE) recommendations. ProFaNE developed a common set of outcome definitions and measures for such future trials. One of the recommendations is to explicitly focus on falls as a primary outcome measure [55].

Based on the results of this review it can be assumed that especially SS-WBV has the potential to provide a small but significant benefit for postural control. It might be that the use of WBV in physical therapy holds promise for those patients or older deconditioned individuals that need to be "skilled-up" for regular training. These are for example frail elderly who cannot perform regular types of exercises, e.g. strength and balance training.

Furthermore, falls have multi-factorial causes [33]. The maintenance of balance depends on the interaction of multiple sensory systems, the motor, and integrative body system. A marked deficit in one of these factors may be sufficient to increase the risk of falling; however, a combination of minor or moderate impairments in multiple physiological domains may also increase the risk of falling. The significant improvement of one factor may be sufficient to decrease the risk of falling.

In addition, we deal with the risk factors of falls. Assessments are used to quantify the risk of falling. In these assessments, certain limit cut-off values are described. If the patient`s balance capacity is below these cut-off values, the risk of falling is increased. Patients, who are slightly above the cut-off values, are only marginally able to improve postural control with WBV, but sufficiently for improvements in daily life.

This review does not allow formulating "best evidence" guidelines for WBV training to improve balance in elderly. However, the review indicates that there are different effects observable between purely vertically and side-alternating WBV systems. The observed effect sizes for these two systems seem to indicate that rather SS-WBV might have potential to influence postural control in elderly. This also because our findings seem to be supported by other reviews on this topic that included studies of albeit lower methodological quality [26,56].

## Conclusion

Two kinds of sinusoidal WBV can be identified for vibration treatments or training sessions that aim to improve balance in elderly. The footplates vibrate either exclusively vertically or in a seesawing manner around a central axis [29]. The question about the effectiveness of this treatment modality cannot be answered conclusively because of several methodological shortcomings and the lack of statistical significance in several outcomes. Therefore, studies with sound methodology and an adequate number of elderly (deconditioned or frail) participants are needed.

## Competing interests

The authors declare that they have no competing interests.

## Authors' contributions

LR initiated the idea for the meta-analysis. KH, RH, SR collected the data. RH conducted the statistical analyses. SR and EDB wrote the paper. KH helped to write the text. LR and EDB supervised the data collection, statistical analysis, and writing of the paper and critically revised the text for its content. All authors have read and approved the final manuscript.

## Pre-publication history

The pre-publication history for this paper can be accessed here:

http://www.biomedcentral.com/1471-2318/11/72/prepub

## Supplementary Material

Additional file 1 **Protocol review**. Click here for file

Additional file 2 **Forest plot of 3 trials comparing the effects of any type of vibration and control interventions on mixed balance**. The analyses were separated for trials reporting post-values (i.e. mean and SD from follow-up) and for trials that reported change values (i.e. mean and SD from the changes from baseline to follow-up). Random effects model with predictive interval. The predictive interval indicates the range within which we expect the effects of 95% of future studies. Values on x-axis denote SMDs. Click here for file

Additional file 3 **Forest plot of 11 trials (12 comparisons comparing) stratified for the vibration type (vertical and side alternating)**. Outcomes were tests for dynamic balance. The analyses were separated for trials reporting post-values (i.e. mean and SD from follow-up) and for trials that reported change values (i.e. mean and SD from the changes from baseline to follow-up). Random effects model with predictive interval. The predictive interval indicates the range within which we expect the effects of 95% of future studies will be. Values on x-axis denote SMDs. Click here for file
