# Supplementary material for: Effects of whole-body vibration on postural control in elderly: a systematic review and meta-analysis
Source: BMC Geriatr. 2011 Nov 3;11:72. doi: 10.1186/1471-2318-11-72 (PMC3229447; doi:10.1186/1471-2318-11-72)
Supplement: Additional file 1 — Protocol review. [file 1471-2318-11-72-S1.DOCX]

**Additional file 1**

**Systematic Review Protocol**

**Effects of whole–body vibration on postural control in elderly: a systematic review and meta-analysis**

Rogan S^1^, Hilfiker R^2^, Herren K^3^, Radlinger L^1^, de Bruin ED^4^

^1^Bern University of Applied Sciences - Health, Bern, Switzerland

^2^University of Applied Sciences Western Switzerland, Leukerbad, Switzerland

^3^ Bern University Hospital, Bern, Switzerland

^4^Institute of Human Movement Sciences and Sport, ETH Zurich, Zurich, Switzerland

Email addresses:

SR: [slavko.rogan@bfh.ch](mailto:slavko.rogan@bfh.ch*)

RH: roger.hilfiker@hevs.ch

HK: kaspar.herren@insel.ch

RL: lorenz.radlinger@bfh.ch

EDB: [eling.debruin@move.biol.ethz.ch](mailto:eling.debruin@move.biol.ethz.ch)*

## *Corresponding author

Index of Contents

[*Corresponding author 2](#_Toc298129189)

[Background 4](#_Toc298129190)

[Research Question 7](#_Toc298129191)

[Methods 8](#_Toc298129192)

[Data Extraction and Quality Assessment 11](#_Toc298129193)

[Time Table 12](#_Toc298129194)

[Appendix 14](#_Toc298129195)

[Pubmed 14](#_Toc298129196)

## Background

Postural control is considered a complex motor skill derived from the interaction of multiple sensorimotor processes. The ability to stand, to walk and to perform daily activities in a safe manner depends on a complex interaction of physiological mechanism.

Many systems need to be evaluated to understand what is wrong with a person´s balance. The sensorimotor control of posture and movement is nowadays often formalized by using feedback loops, in imitation of technical systems (Norbert Wiener). Basic principle usually represents a system consisting of a "regulator" (the CNS), based on a "regulatory" (controlled system, supporting structures) and thereby influences creates a "control variable" (the balance"). Sensors on the controlled variable is fed back and compared with a setpoint. This is also the basis for recent observations of the static and dynamic balance control.

It is widely recognized that with increasing age, patients with for Parkinson disease, multiple sclerosis, and stroke; and athletes with balance disorders, suffer from risk of injury or risk of falling. Falls are multifactorial in origin.

For Healthy older persons for example, have measurable declines in each sensory system related to balance. Touch/pressure sensation on plantar surface, joint position sense, visual acuity, visual edge detection, and vestibular input are reduced in advanced age and are associated with decrements in laboratory measures of balance. In addition, it can be generally observed that dizziness, physiological changes in muscle strength, proprioception and loss of reaction favoring falls. In older persons, for example, five percent of falls to suffer fractures. Another ten to fifteen percent are left with severe injuries. Balance related problems are result in considerable health and social services costs because of loss of confidence, injury inability to maintain a safe environment, and dependence in basic functional activities.

Due to the influence of WBV on the neuromuscular system and the triggering of reflex responses, mechanical vibrations have the potential to be used in treatment to prevent loss of balance. This is especially true for individual persons or patients who have limited or altered reflex generation.

It has been hypothesized, that transmission of vibrations to a biological system can lead to physiological changes in numerous levels: Stimulation of subcutaneous proprioceptors, which influence the γ-loop, increasing/decreasing muscle spindle sensitivity. WBV probably activates muscle spindle activity to cause muscle contraction via α-pathways, may activate Golgi tendon organs and thus muscle such as tonic vibration reflexes and even antagonistic vibration reflexes.

Furthermore, WBV stimulates the vestibular systems; changes in cerebral activity, e.g. in the thalamus and somatosensory cortex, changes of neurotransmitter concentration such as those in dopamine and serotonine, and changes in hormone concentrations, have been described.

In addition to the direction and the vibration character, there are several treatment parameters that are important to consider when using WBV. These include frequency, amplitude (mm), the length of an impulse (sec.), the amount of an impulse (series), the density of the break (sec.), frequency of training (times per week) and the duration of the training.

Most commonly, WBV studies have used frequencies ranging from 25-50Hz, amplitudes from 2-10mm and total duration of 30 sec -10 min. The methodological design of an effective vibration training is still insufficiently investigated until now and is based mostly on experience.

Therefore, physiotherapists need ways for treatment the people with WBV.

Whole - body vibration exerts a substantive influence to improve strength, balance or bone density. The aim of this present paper was to ascertain the effect of whole-body on the postural control in human.

## Research Question

The objective of this review is to establish the best available evidence regarding whole-body vibration training in order to postural control in humans until September 2011. Specifically, the review will determine:

- Are there variations in the static, dynamic and functional balance after WBV training?

## Methods

Searching Desing

We followed the PRISMA guidelines (http://www.prisma-statement.org/).

The search strategy aims to find published studies and papers. Electronic search of the following databases until September 2010:

- PubMed Cochrane
- PEDro
- Cinahl (Ebsco Host).
- Furthermore, we inspected the reference lists of all retrieved studies.

MESH terms as aging, postural balance were used for the identification of relevant studies.

PubMed

((((((((((((((aged[Mesh:noexp])) OR (elderly)) OR (older people)) OR (dwelling home)) OR (nursing home)) OR (elderly women)) OR (postmenopausal)) OR (frail elderly[Mesh:noexp])) OR (aging[Mesh:noexp]))) AND (((((((((wbv)) OR (wbv AND exposure)) OR (whole body vibration*)) OR (whole-body vibration*)) OR (random vibration)) OR (stochastic vibration)) OR (vibration training)) OR (vibration noise))) AND (((((((((((aged[Mesh:noexp])) OR (elderly)) OR (older people)) OR (dwelling home)) OR (nursing home)) OR (elderly women)) OR (postmenopausal)) OR (frail elderly[Mesh:noexp])) OR (aging[Mesh:noexp]))) AND (((((((((wbv)) OR (wbv AND exposure)) OR (whole body vibration*)) OR (whole-body vibration*)) OR (random vibration)) OR (stochastic vibration)) OR (vibration training)) OR (vibration noise)))) AND (((((((((((((postural control)) OR (balance)) OR (postural stability)) OR (postural balance[Mesh])) OR (sensomotoric)) OR (senso motoric)) OR (dynamic stability)) OR (static balance)) OR (dynamic balance)) OR (functional balance)) OR (fall)) OR (falls))

Cochrane Central , Pedro , Cinahl :

(Whole body vibration) OR (whole-body vibration) OR (whole-body-vibration) OR (whole body vibrations) OR (stochastic resonance) or (stochastic vibration) or (stochastic vibrations) OR (stochastic training) OR (stochastic therapy) OR (vibration therapy) OR (vibration treatment) OR (vibration training)

AND

balance OR ("postural control") OR (postural stability) OR (postural reaction) OR (equilib*) OR (sway) OR (fall) OR (falls) OR (faller) OR (fallers) OR (gait stability) OR (dynamic stability) OR (postural stead*) OR (sensorimotor) OR (sensori-motor) OR (proprioception) OR (proprioceptive)

## Data Extraction and Quality Assessment

The methodological quality was independently assessed by three reviewers.

Scored with “The Cochrane Collaboration’s tool for assessing risk of bias“. The criteria list comprised six items. Each item was scored “+” if the criterion was fulfilled, “-“ if the criterion was not fulfilled, and “?” if the information was not provided or was unclear.

One author (S.R.) independently abstracted the following information from each of the studies included in this review: 1) design and sample; 2) inclusion criteria; 3) type of intervention; 4) type of WBV system; 5) change in static, dynamic and functional balance parameters; and 6) conclusions from the studies and statistical significance.

Heterogeneity was assessed by examining forest plots and the I^2^ statistics.

We using the standardized mean difference (SMD) as effect measures, and presented as continuous data (mean values and SD or mean changes). Magnitude of the effect size, calculated by SMD, where d>0.2-0.5 indicates a small effect size, d>0.5-0.8 indicates a medium effect size, d>0.8 indicates a large effect size. For publication bias we using funnel plot.

## Time Table

| Date | Work | Author |
| --- | --- | --- |
| 01.2010 | Management systematic review | LR, RH, KH, SR |
| Till 01.2010 | Search Database: Pubmed, Cinhal, Cochrane, PEDRO, Google Scholar  Private Database | RH  SR |
| Till 01.2010 | Evaluate Abstracts | RH, KH, SR |
| Till 05.2010 | Read full Text | RH, KH, SR |
| Till 08.2010 | Evaluate full Text: risk of Bias, Outcome Table | RH, KH, SR |
| Till 10.2010 | Collect Data, analyses Data | RH, EDB |
| Till 12.2010 | Writing Review | SR, KH, RH, EDB |
| 6.2011 | Proofread English | Flying Teacher |
| 7.2011 | Final check: manuscript, Prisma Checklist, Flow Chart, Copyright Transfer | SR, EDB |
| Till.11.7.2011 | Submitted manuscript BMC Geriatric | EDB |

## Appendix

### Pubmed

1.

[Effect of whole body vibration in Parkinson's disease: a controlled study.](http://www.ncbi.nlm.nih.gov/pubmed/19199362)

Arias P, Chouza M, Vivas J, Cudeiro J.

Mov Disord. 2009 Apr 30;24(6):891-8.

2.

[The feasibility of Whole Body Vibration in institutionalised elderly persons and its influence on muscle performance, balance and mobility: a randomised controlled trial [ISRCTN62535013].](http://www.ncbi.nlm.nih.gov/pubmed/16372905)

Bautmans I, Van Hees E, Lemper JC, Mets T.

BMC Geriatr. 2005 Dec 22;5:17.

3.

[The effect of 8 mos of twice-weekly low- or higher intensity whole body vibration on risk factors for postmenopausal hip fracture.](http://www.ncbi.nlm.nih.gov/pubmed/21403595)

Beck BR, Norling TL.

Am J Phys Med Rehabil. 2010 Dec;89(12):997-1009.

4.

[Changes in balance, functional performance and fall risk following whole body vibration training and vitamin D supplementation in institutionalized elderly women. A 6 month randomized controlled trial.](http://www.ncbi.nlm.nih.gov/pubmed/21256028)

Bogaerts A, Delecluse C, Boonen S, Claessens AL, Milisen K, Verschueren SM.

Gait Posture. 2011 Mar;33(3):466-72. Epub 2011 Jan 20.

5.

[Effects of whole body vibration training on postural control in older individuals: a 1 year randomized controlled trial.](http://www.ncbi.nlm.nih.gov/pubmed/17074485)

Bogaerts A, Verschueren S, Delecluse C, Claessens AL, Boonen S.

Gait Posture. 2007 Jul;26(2):309-16. Epub 2006 Oct 30.

6.

[Effects of whole body vibration training on cardiorespiratory fitness and muscle strength in older individuals (a 1-year randomised controlled trial).](http://www.ncbi.nlm.nih.gov/pubmed/19439517)

Bogaerts AC, Delecluse C, Claessens AL, Troosters T, Boonen S, Verschueren SM.

Age Ageing. 2009 Jul;38(4):448-54. Epub 2009 May 13.

7.

[Self-reported low back symptoms in urban bus drivers exposed to whole-body vibration.](http://www.ncbi.nlm.nih.gov/pubmed/1411756)

Bovenzi M, Zadini A.

Spine (Phila Pa 1976). 1992 Sep;17(9):1048-59.

8.

[Exploring the effects of a 20-week whole-body vibration training programme on leg muscle performance and function in persons with multiple sclerosis.](http://www.ncbi.nlm.nih.gov/pubmed/20878048)

Broekmans T, Roelants M, Alders G, Feys P, Thijs H, Eijnde BO.

J Rehabil Med. 2010 Oct;42(9):866-72.

9.

[Proprioceptive weighting changes in persons with low back pain and elderly persons during upright standing.](http://www.ncbi.nlm.nih.gov/pubmed/15265591)

Brumagne S, Cordo P, Verschueren S.

Neurosci Lett. 2004 Aug 5;366(1):63-6.

10.

[Controlled whole body vibration to decrease fall risk and improve health-related quality of life of nursing home residents.](http://www.ncbi.nlm.nih.gov/pubmed/15706558)

Bruyere O, Wuidart MA, Di Palma E, Gourlay M, Ethgen O, Richy F, Reginster JY.

Arch Phys Med Rehabil. 2005 Feb;86(2):303-7.

11.

[Vibratory thresholds and mobility in older persons.](http://www.ncbi.nlm.nih.gov/pubmed/19306325)

Buchman AS, Wilson RS, Leurgans S, Bennett DA.

Muscle Nerve. 2009 Jun;39(6):754-60.

12.

[Postural load and back pain of workers in the manufacturing of prefabricated concrete elements.](http://www.ncbi.nlm.nih.gov/pubmed/1833181)

Burdorf A, Govaert G, Elders L.

Ergonomics. 1991 Jul;34(7):909-18.

13.

[Does whole-body vibration training have acute residual effects on postural control ability of elderly women?](http://www.ncbi.nlm.nih.gov/pubmed/21088549)

Carlucci F, Mazzà C, Cappozzo A.

J Strength Cond Res. 2010 Dec;24(12):3363-8.

14.

[High-frequency whole-body vibration improves balancing ability in elderly women.](http://www.ncbi.nlm.nih.gov/pubmed/17601464)

Cheung WH, Mok HW, Qin L, Sze PC, Lee KM, Leung KS.

Arch Phys Med Rehabil. 2007 Jul;88(7):852-7.

15.

[Whole body vibration versus conventional physiotherapy to improve balance and gait in Parkinson's disease.](http://www.ncbi.nlm.nih.gov/pubmed/18295614)

Ebersbach G, Edler D, Kaufhold O, Wissel J.

Arch Phys Med Rehabil. 2008 Mar;89(3):399-403.

16.

[Reaction and movement times in men of different ages: a population study.](http://www.ncbi.nlm.nih.gov/pubmed/3748725)

Era P, Jokela J, Heikkinen E.

Percept Mot Skills. 1986 Aug;63(1):111-30.

17.

[Controlling balance decline across the menopause using a balance-strategy training program: a randomized, controlled trial.](http://www.ncbi.nlm.nih.gov/pubmed/19058060)

Fu S, Choy NL, Nitz J.

Climacteric. 2009 Apr;12(2):165-76.

18.

[Efficacy of a whole-body vibration intervention on functional performance of community-dwelling older adults.](http://www.ncbi.nlm.nih.gov/pubmed/20590479)

Furness TP, Maschette WE, Lorenzen C, Naughton GA, Williams MD.

J Altern Complement Med. 2010 Jul;16(7):795-7.

19.

[Influence of whole body vibration platform frequency on neuromuscular performance of community-dwelling older adults.](http://www.ncbi.nlm.nih.gov/pubmed/19620913)

Furness TP, Maschette WE.

J Strength Cond Res. 2009 Aug;23(5):1508-13.

20.

[Subsensory vibrations to the feet reduce gait variability in elderly fallers.](http://www.ncbi.nlm.nih.gov/pubmed/19632845)

Galica AM, Kang HG, Priplata AA, D'Andrea SE, Starobinets OV, Sorond FA, Cupples LA, Lipsitz LA.

Gait Posture. 2009 Oct;30(3):383-7. Epub 2009 Jul 25.

21.

[Effects of vibratory orthosis on balance in idiopathic Parkinson's disease.](http://www.ncbi.nlm.nih.gov/pubmed/19172481)

Ghoseiri K, Forogh B, Sanjari MA, Bavi A.

Disabil Rehabil Assist Technol. 2009 Jan;4(1):58-63.

22.

[[Whole-body vibration training: fact or fiction?].](http://www.ncbi.nlm.nih.gov/pubmed/18777735)

Gojanovic B, Gremion G, Waeber B.

Rev Med Suisse. 2008 Aug 6;4(166):1712-6.

23.

[Tilt vibratory exercise and the dynamic balance in fibromyalgia: A randomized controlled trial.](http://www.ncbi.nlm.nih.gov/pubmed/20235191)

Gusi N, Parraca JA, Olivares PR, Leal A, Adsuar JC.

Arthritis Care Res (Hoboken). 2010 Aug;62(8):1072-8.

24.

[Low-frequency vibratory exercise reduces the risk of bone fracture more than walking: a randomized controlled trial.](http://www.ncbi.nlm.nih.gov/pubmed/17137514)

Gusi N, Raimundo A, Leal A.

BMC Musculoskelet Disord. 2006 Nov 30;7:92.

25.

[Proprioceptive and sensorimotor performance in Parkinson's disease.](http://www.ncbi.nlm.nih.gov/pubmed/17214404)

Haas CT, Buhlmann A, Turbanski S, Schmidtbleicher D.

Res Sports Med. 2006 Oct-Dec;14(4):273-87.

26.

[Muscular and neurologic function in patients with recurrent dislocation after total hip arthroplasty: a matched controlled study of 65 patients using dual-energy X-ray absorptiometry and postural stability tests.](http://www.ncbi.nlm.nih.gov/pubmed/10220186)

Hedlundh U, Karlsson M, Ringsberg K, Besjakov J, Fredin H.

J Arthroplasty. 1999 Apr;14(3):319-25.

27.

[Development of vibrating insoles.](http://www.ncbi.nlm.nih.gov/pubmed/17975456)

Hijmans JM, Geertzen JH, Schokker B, Postema K.

Int J Rehabil Res. 2007 Dec;30(4):343-5.

28.

[Effects of vibrating insoles on standing balance in diabetic neuropathy.](http://www.ncbi.nlm.nih.gov/pubmed/19319766)

Hijmans JM, Geertzen JH, Zijlstra W, Hof AL, Postema K.

J Rehabil Res Dev. 2008;45(9):1441-9.

29.

[Smoking habits and postural stability.](http://www.ncbi.nlm.nih.gov/pubmed/8179103)

Iki M, Ishizaki H, Aalto H, Starck J, Pyykkö I.

Am J Otolaryngol. 1994 Mar-Apr;15(2):124-8.

30.

[Vibration-induced white finger as a risk factor for hearing loss and postural instability.](http://www.ncbi.nlm.nih.gov/pubmed/7708096)

Iki M.

Nagoya J Med Sci. 1994 May;57 Suppl:137-45.

31.

[Effect of whole-body vibration exercise on lumbar bone mineral density, bone turnover, and chronic back pain in post-menopausal osteoporotic women treated with alendronate.](http://www.ncbi.nlm.nih.gov/pubmed/15977465)

Iwamoto J, Takeda T, Sato Y, Uzawa M.

Aging Clin Exp Res. 2005 Apr;17(2):157-63.

32.

[Salient and placebo vibrotactile feedback are equally effective in reducing sway in bilateral vestibular loss patients.](http://www.ncbi.nlm.nih.gov/pubmed/19926286)

Janssen M, Stokroos R, Aarts J, van Lummel R, Kingma H.

Gait Posture. 2010 Feb;31(2):213-7. Epub 2009 Nov 18.

33.

[Physical therapy approaches to reduce fall and fracture risk among older adults.](http://www.ncbi.nlm.nih.gov/pubmed/20517287)

Karinkanta S, Piirtola M, Sievänen H, Uusi-Rasi K, Kannus P.

Nat Rev Endocrinol. 2010 Jul;6(7):396-407. Epub 2010 Jun 1. Review.

34.

[Head movement restriction and postural stability in patients with compensated unilateral vestibular loss.](http://www.ncbi.nlm.nih.gov/pubmed/9821908)

Karlberg M, Magnusson M.

Arch Phys Med Rehabil. 1998 Nov;79(11):1448-50.

35.

[Effect of whole-body vibration exercise and muscle strengthening, balance, and walking exercises on walking ability in the elderly.](http://www.ncbi.nlm.nih.gov/pubmed/17392595)

Kawanabe K, Kawashima A, Sashimoto I, Takeda T, Sato Y, Iwamoto J.

Keio J Med. 2007 Mar;56(1):28-33.

36.

[[Effect of whole body vibration on the neuromuscular performance of females 65 years and older. One-year results of the controlled randomized ELVIS study].](http://www.ncbi.nlm.nih.gov/pubmed/19789832)

Kemmler W, V Stengel S, Mayer S, Niedermayer M, Hentschke C, Kalender WA.

Z Gerontol Geriatr. 2010 Apr;43(2):125-32. Epub 2009 Oct 1. German.

37.

[Gamma loop dysfunction of the quadriceps femoris of elderly patients hospitalized after fall injury.](http://www.ncbi.nlm.nih.gov/pubmed/18171488)

Konishi Y, Kasukawa T, Tobita H, Nishino A, Konishi M.

J Geriatr Phys Ther. 2007;30(2):54-9.

38.

[Asymmetric vestibular function in the elderly might be a significant contributor to hip fractures.](http://www.ncbi.nlm.nih.gov/pubmed/10853718)

Kristinsdottir EK, Jarnlo GB, Magnusson M.

Scand J Rehabil Med. 2000 Jun;32(2):56-60.

39.

["The vibrometer". An electro magnetic transducer as an attempt to examine sensibility of the hand in quantitative terms.](http://www.ncbi.nlm.nih.gov/pubmed/7286809)

Mansat M, Delprat J, Delprat JM.

Hand. 1981 Jun;13(2):202-10.

40.

[[Occupational changes in the organ of hearing and balance in sailors and fisherman].](http://www.ncbi.nlm.nih.gov/pubmed/6966436)

Meniakin RP, Poperetskaia VI.

Vestn Otorinolaringol. 1980 Jan-Feb;(1):39-43. Russian. No abstract available.

41.

[Combined whole body vibration and balance training using Vibrosphere® : Improvement of trunk stability, muscle tone, and postural control in stroke patients during early geriatric rehabilitation.](http://www.ncbi.nlm.nih.gov/pubmed/21505939)

Merkert J, Butz S, Nieczaj R, Steinhagen-Thiessen E, Eckardt R.

Z Gerontol Geriatr. 2011 Apr 21. [Epub ahead of print]

42.

[The effects of whole-body vibration training in aging adults: a systematic review.](http://www.ncbi.nlm.nih.gov/pubmed/20128338)

Merriman H, Jackson K.

J Geriatr Phys Ther. 2009;32(3):134-45. Review.

43.

[Balancing with vibration: a prelude for "drift and act" balance control.](http://www.ncbi.nlm.nih.gov/pubmed/19841741)

Milton JG, Ohira T, Cabrera JL, Fraiser RM, Gyorffy JB, Ruiz FK, Strauss MA, Balch EC, Marin PJ, Alexander JL.

PLoS One. 2009 Oct 20;4(10):e7427.

44.

[Are clinical measurements of uncomfortable loudness levels a valid indicator of real-world auditory discomfort?](http://www.ncbi.nlm.nih.gov/pubmed/9845026)

Munro KJ, Patel RK.

Br J Audiol. 1998 Oct;32(5):287-93.

45.

[Is the Wii Fit a new-generation tool for improving balance, health and well-being? A pilot study.](http://www.ncbi.nlm.nih.gov/pubmed/19905991)

Nitz JC, Kuys S, Isles R, Fu S.

Climacteric. 2010 Oct;13(5):487-91.

46.

[Change of body movement coordination during cervical proprioceptive disturbances with increased age.](http://www.ncbi.nlm.nih.gov/pubmed/20016118)

Patel M, Fransson PA, Karlberg M, Malmstrom EM, Magnusson M.

Gerontology. 2010;56(3):284-90. Epub 2009 Dec 10.

47.

[Adaptation and vision change the relationship between muscle activity of the lower limbs and body movement during human balance perturbations.](http://www.ncbi.nlm.nih.gov/pubmed/19136294)

Patel M, Gomez S, Lush D, Fransson PA.

Clin Neurophysiol. 2009 Mar;120(3):601-9. Epub 2009 Jan 10.

48.

[Evaluation of age-related plantar-surface insensitivity and onset age of advanced insensitivity in older adults using vibratory and touch sensation tests.](http://www.ncbi.nlm.nih.gov/pubmed/16183200)

Perry SD.

Neurosci Lett. 2006 Jan 9;392(1-2):62-7. Epub 2005 Sep 23.

49.

[[Vibrational physical exercises as the rehabilitation in gerontology].](http://www.ncbi.nlm.nih.gov/pubmed/19947400)

Piatin VF, Shirolapov IV, Nikitin OL.

Adv Gerontol. 2009;22(2):337-42. Russian.

50.

[Effects of local vibrations on skeletal muscle trophism in elderly people: mechanical, cellular, and molecular events.](http://www.ncbi.nlm.nih.gov/pubmed/19724891)

Pietrangelo T, Mancinelli R, Toniolo L, Cancellara L, Paoli A, Puglielli C, Iodice P, Doria C, Bosco G, D'Amelio L, di Tano G, Fulle S, Saggini R, Fanò G, Reggiani C.

Int J Mol Med. 2009 Oct;24(4):503-12.

51.

[Muscle activity and acceleration during whole body vibration: effect of frequency and amplitude.](http://www.ncbi.nlm.nih.gov/pubmed/20541297)

Pollock RD, Woledge RC, Mills KR, Martin FC, Newham DJ.

Clin Biomech (Bristol, Avon). 2010 Oct;25(8):840-6. Epub 2010 Jun 11.

52.

[Vibrating insoles and balance control in elderly people.](http://www.ncbi.nlm.nih.gov/pubmed/14550702)

Priplata AA, Niemi JB, Harry JD, Lipsitz LA, Collins JJ.

Lancet. 2003 Oct 4;362(9390):1123-4.

53.

[Noise-enhanced balance control in patients with diabetes and patients with stroke.](http://www.ncbi.nlm.nih.gov/pubmed/16287079)

Priplata AA, Patritti BL, Niemi JB, Hughes R, Gravelle DC, Lipsitz LA, Veves A, Stein J, Bonato P, Collins JJ.

Ann Neurol. 2006 Jan;59(1):4-12.

54.

[Effects of whole body vibration on the skeleton and other organ systems in man and animal models: what we know and what we need to know.](http://www.ncbi.nlm.nih.gov/pubmed/18762281)

Prisby RD, Lafage-Proust MH, Malaval L, Belli A, Vico L.

Ageing Res Rev. 2008 Dec;7(4):319-29. Epub 2008 Aug 12. Review.

55.

[Does impulse noise induce vestibular disturbances?](http://www.ncbi.nlm.nih.gov/pubmed/2635505)

Pyykkö I, Aalto H, Ylikoski J.

Acta Otolaryngol Suppl. 1989;468:211-6.

56.

[Fitness efficacy of vibratory exercise compared to walking in postmenopausal women.](http://www.ncbi.nlm.nih.gov/pubmed/19434420)

Raimundo AM, Gusi N, Tomas-Carus P.

Eur J Appl Physiol. 2009 Jul;106(5):741-8. Epub 2009 May 12.

57.

[[What can we think about whole-body-vibration in elderly people?].](http://www.ncbi.nlm.nih.gov/pubmed/20570086)

Raschilas F, Blain H.

Presse Med. 2010 Oct;39(10):1032-7. Epub 2010 May 31. Review.

58.

[Effects of vibration exercise on muscle performance and mobility in an older population.](http://www.ncbi.nlm.nih.gov/pubmed/18048942)

Rees S, Murphy A, Watsford M.

J Aging Phys Act. 2007 Oct;15(4):367-81.

59.

[Effects of whole body vibration on postural steadiness in an older population.](http://www.ncbi.nlm.nih.gov/pubmed/18550436)

Rees SS, Murphy AJ, Watsford ML.

J Sci Med Sport. 2009 Jul;12(4):440-4. Epub 2008 Jun 11.

60.

[The effects of stochastic resonance stimulation on spine proprioception and postural control in chronic low back pain patients.](http://www.ncbi.nlm.nih.gov/pubmed/19214090)

Reeves NP, Cholewicki J, Lee AS, Mysliwiec LW.

Spine (Phila Pa 1976). 2009 Feb 15;34(4):316-21.

61.

[Vibration as an exercise modality: how it may work, and what its potential might be.](http://www.ncbi.nlm.nih.gov/pubmed/20012646)

Rittweger J.

Eur J Appl Physiol. 2010 Mar;108(5):877-904. Epub 2009 Dec 12. Review.

62.

[Motor control in humans with large-fiber sensory neuropathy.](http://www.ncbi.nlm.nih.gov/pubmed/2993208)

Sanes JN, Mauritz KH, Dalakas MC, Evarts EV.

Hum Neurobiol. 1985;4(2):101-14.

63.

[Effects of whole-body vibration in patients with multiple sclerosis: a pilot study.](http://www.ncbi.nlm.nih.gov/pubmed/16323382)

Schuhfried O, Mittermaier C, Jovanovic T, Pieber K, Paternostro-Sluga T.

Clin Rehabil. 2005 Dec;19(8):834-42.

64.

[[Physiological mechanisms fo the formation of the adaptation- compensatory process with the impact of local vibration accompanied by noise and dust].](http://www.ncbi.nlm.nih.gov/pubmed/10741062)

Shevtsova VM.

Med Tr Prom Ekol. 2000;(2):18-23. Russian.

65.

[Vibration effects on static balance and strength.](http://www.ncbi.nlm.nih.gov/pubmed/20589590)

Spiliopoulou SI, Amiridis IG, Tsigganos G, Economides D, Kellis E.

Int J Sports Med. 2010 Sep;31(9):610-6. Epub 2010 Jun 29.

66.

[Whole body vibration compared to conventional physiotherapy in patients with gonarthrosis: a protocol for a randomized, controlled study.](http://www.ncbi.nlm.nih.gov/pubmed/20565956)

Stein G, Knoell P, Faymonville C, Kaulhausen T, Siewe J, Otto C, Eysel P, Zarghooni K.

BMC Musculoskelet Disord. 2010 Jun 21;11:128.

67.

[Air versus bone conduction: an equal loudness investigation.](http://www.ncbi.nlm.nih.gov/pubmed/12117525)

Stenfelt S, Håkansson B.

Hear Res. 2002 May;167(1-2):1-12.

68.

[[Effects of exposure to occupational hand-arm vibration on maintenance of postural balance].](http://www.ncbi.nlm.nih.gov/pubmed/15656083)

Tanaka K, Maeda T, Tanaka M, Fukushima T.

Sangyo Eiseigaku Zasshi. 2004 Nov;46(6):223-8. Japanese.

69.

[Effect of four-month vertical whole body vibration on performance and balance.](http://www.ncbi.nlm.nih.gov/pubmed/12218749)

Torvinen S, Kannus P, Sievänen H, Järvinen TA, Pasanen M, Kontulainen S, Järvinen TL, Järvinen M, Oja P, Vuori I.

Med Sci Sports Exerc. 2002 Sep;34(9):1523-8.

70.

[Effect of 4-min vertical whole body vibration on muscle performance and body balance: a randomized cross-over study.](http://www.ncbi.nlm.nih.gov/pubmed/12165890)

Torvinen S, Sievänen H, Järvinen TA, Pasanen M, Kontulainen S, Kannus P.

Int J Sports Med. 2002 Jul;23(5):374-9.

71.

[Effect of whole body vibration exercise on muscle strength and proprioception in females with knee osteoarthritis.](http://www.ncbi.nlm.nih.gov/pubmed/19147365)

Trans T, Aaboe J, Henriksen M, Christensen R, Bliddal H, Lund H.

Knee. 2009 Aug;16(4):256-61. Epub 2009 Jan 15.

72.

[Correlations between certain hearing changes and vegetative balance in miners.](http://www.ncbi.nlm.nih.gov/pubmed/15068211)

Tsaneva L, Dukov R.

Cent Eur J Public Health. 2004 Mar;12(1):49-52.

73.

[Effects of random whole-body vibration on postural control in Parkinson's disease.](http://www.ncbi.nlm.nih.gov/pubmed/16392539)

Turbanski S, Haas CT, Schmidtbleicher D, Friedrich A, Duisberg P.

Res Sports Med. 2005 Jul-Sep;13(3):243-56.

74.

[Audiological problems in patients with tinnitus exposed to noise and vibrations.](http://www.ncbi.nlm.nih.gov/pubmed/11125978)

Tzaneva L, Savov A, Damianova V.

Cent Eur J Public Health. 2000 Nov;8(4):233-5.

75.

[Short-term effects of whole-body vibration on postural control in unilateral chronic stroke patients: preliminary evidence.](http://www.ncbi.nlm.nih.gov/pubmed/15502741)

van Nes IJ, Geurts AC, Hendricks HT, Duysens J.

Am J Phys Med Rehabil. 2004 Nov;83(11):867-73.

76.

[Long-term effects of 6-week whole-body vibration on balance recovery and activities of daily living in the postacute phase of stroke: a randomized, controlled trial.](http://www.ncbi.nlm.nih.gov/pubmed/16902175)

van Nes IJ, Latour H, Schils F, Meijer R, van Kuijk A, Geurts AC.

Stroke. 2006 Sep;37(9):2331-5. Epub 2006 Aug 10.

77.

[Effect of 6-month whole body vibration training on hip density, muscle strength, and postural control in postmenopausal women: a randomized controlled pilot study.](http://www.ncbi.nlm.nih.gov/pubmed/15040822)

Verschueren SM, Roelants M, Delecluse C, Swinnen S, Vanderschueren D, Boonen S.

J Bone Miner Res. 2004 Mar;19(3):352-9. Epub 2003 Dec 22.

78.

[Modeling the effect of channel number and interaction on consonant recognition in a cochlear implant peak-picking strategy.](http://www.ncbi.nlm.nih.gov/pubmed/19275329)

Verschuur C.

J Acoust Soc Am. 2009 Mar;125(3):1723-36.

79.

[Auditory localization abilities in bilateral cochlear implant recipients.](http://www.ncbi.nlm.nih.gov/pubmed/16151344)

Verschuur CA, Lutman ME, Ramsden R, Greenham P, O'Driscoll M.

Otol Neurotol. 2005 Sep;26(5):965-71.

80.

[Assessing the effect of vibrotactile feedback during continuous multidirectional platform motion: a frequency domain approach.](http://www.ncbi.nlm.nih.gov/pubmed/19964454)

Vichare VV, Wall C, Balkwill MD, Sienko MD.

Conf Proc IEEE Eng Med Biol Soc. 2009;2009:6910-3.

81.

[Speech recognition in noise as a function of highpass-filter cutoff frequency for people with and without low-frequency cochlear dead regions.](http://www.ncbi.nlm.nih.gov/pubmed/18273950)

Vinay, Baer T, Moore BC.

J Acoust Soc Am. 2008 Feb;123(2):606-9.

82.

[Effects of whole body vibration on bone mineral density and falls: results of the randomized controlled ELVIS study with postmenopausal women.](http://www.ncbi.nlm.nih.gov/pubmed/20306017)

von Stengel S, Kemmler W, Engelke K, Kalender WA.

Osteoporos Int. 2011 Jan;22(1):317-25. Epub 2010 Mar 20.

83.

[[Effect of whole body vibration exercise on osteoporotic risk factors].](http://www.ncbi.nlm.nih.gov/pubmed/19603365)

von Stengel S, Kemmler W, Mayer S, Engelke K, Klarner A, Kalender WA.

Dtsch Med Wochenschr. 2009 Jul;134(30):1511-6. Epub 2009 Jul 14. German.

84.

[Vibrotactile tilt feedback improves dynamic gait index: a fall risk indicator in older adults.](http://www.ncbi.nlm.nih.gov/pubmed/19345107)

Wall C 3rd, Wrisley DM, Statler KD.

Gait Posture. 2009 Jul;30(1):16-21. Epub 2009 Apr 2.

85.

[Effect of whole-body vibration on bone properties in aging mice.](http://www.ncbi.nlm.nih.gov/pubmed/20638490)

Wenger KH, Freeman JD, Fulzele S, Immel DM, Powell BD, Molitor P, Chao YJ, Gao HS, Elsalanty M, Hamrick MW, Isales CM, Yu JC.

Bone. 2010 Oct;47(4):746-55. Epub 2010 Jul 16.

86.

[Sensory-specific balance training in older adults: effect on proprioceptive reintegration and cognitive demands.](http://www.ncbi.nlm.nih.gov/pubmed/17636154)

Westlake KP, Culham EG.

Phys Ther. 2007 Oct;87(10):1274-83. Epub 2007 Jul 17.

87.

[Haemodynamic and haemorheological effects of hypervolaemic haemodilution in men with primary hypertension.](http://www.ncbi.nlm.nih.gov/pubmed/3302038)

Wysocki M, Persson B, Aurell M, Braide M, Bagge U, Andersson OK.

J Hypertens. 1987 Apr;5(2):185-9.

88.

[Voice analysis and videolaryngostroboscopy in patients with Parkinson's disease.](http://www.ncbi.nlm.nih.gov/pubmed/12115074)

Yücetürk AV, Yilmaz H, Eğrilmez M, Karaca S.

Eur Arch Otorhinolaryngol. 2002 Jul;259(6):290-3. Epub 2002 Apr 24.

89.

Noise-enhanced human sensorimotor function.

Collins JJ, Priplata AA, Gravelle DC, Niemi J, Harry J, Lipsitz LA.

IEEE Eng Med Biol Mag 22(2): 76-83.

90.

Noise in human muscle spindles.

Cordo P, Inglis JT, Verschueren S, Collins JJ, Merfeld DM, Rosenblum S, Buckley S, Moss F.

Nature. 1996 Oct 31;383(6603):769-70.

91.

Noise-enhanced tactile sensation.

Collins JJ, Imhoff TT, Grigg P.

Nature. 1996 Oct 31;383(6603):770.

92.

Noise-enhanced vibrotactile sensitivity in older adults, patients with stroke, and patients with diabetic neuropathy.

Liu W, Lipsitz LA, Montero-Odasso M, Bean J, Kerrigan DC, Collins JJ.

Arch Phys Med Rehabil. 2002 Feb;83(2):171-6.

93.

Enhancing tactile sensation in older adults with electrical noise stimulation.

Dhruv NT, Niemi JB, Harry JD, Lipsitz LA, Collins JJ.

Neuroreport. 2002 Apr 16;13(5):597-600.

94.

Noise-enhanced balance control in older adults.

Gravelle DC, Laughton CA, Dhruv NT, Katdare KD, Niemi JB, Lipsitz LA, Collins JJ.

Neuroreport. 2002 Oct 28;13(15):1853-6.

95.

Noise-enhanced human balance control.

Priplata A, Niemi J, Salen M, Harry J, Lipsitz LA, Collins JJ.

Phys Rev Lett. 2002 Dec 2;89(23):238101. Epub 2002 Nov 13. The Cochrane Collaboration’s tool for assessing risk of bias Table

| Domain | Description | Review authors’ judgement |
| --- | --- | --- |
| Sequence generation. | Describe the method used to generate the allocation sequence in sufficient detail to allow an assessment of whether it should produce comparable groups. | Was the allocation sequence adequately generated? |
| Allocation concealment. | Describe the method used to conceal the allocation sequence in sufficient detail to determine whether intervention allocations could have been foreseen in advance of, or during, enrolment. | Was allocation adequately concealed? |
| Blinding of participants, personnel and outcome assessors Assessments should be made for each main outcome (or class of outcomes). | Describe all measures used, if any, to blind study participants and personnel from knowledge of which intervention a participant received. Provide any information relating to whether the intended blinding was effective. | Was knowledge of the allocated intervention adequately prevented during the study? |
| Incomplete outcome data Assessments should be made for each main outcome (or class of outcomes). | Describe the completeness of outcome data for each main outcome, including attrition and exclusions from the analysis. State whether attrition and exclusions were reported, the numbers in each intervention group (compared with total randomized participants), reasons for attrition/exclusions where reported, and any re-inclusions in analyses performed by the review authors. | Were incomplete outcome data adequately addressed? |
| Selective outcome reporting. | State how the possibility of selective outcome reporting was examined by the review authors, and what was found. | Are reports of the study free of suggestion of selective outcome reporting? |
| Other sources of bias. | State any important concerns about bias not addressed in the other domains in the tool.  If particular questions/entries were pre-specified in the review’s protocol, responses should be provided for each question/entry. | Was the study apparently free of other problems that could put it at a high risk of bias? |

 The description

The description provides a succinct summary from which judgements of risk of bias can be made, and aims to ensure transparency in how these judgements are reached. For a specific study, information for the description will often come from a single published study report, but may be obtained from a mixture of study reports, protocols, published comments on the study and contacts with the investigators. Where appropriate, the description should include verbatim quotes from reports or correspondence. A helpful construction to supplement an ambiguous quote is to state ‘Probably done’ or ‘Probably not done’, providing the rationale for such assertions. When no information is available from which to make a judgement, this should be stated explicitly.

Examples of summary descriptions for Sequence generation entry

| Sequence generation. | Comment: no information provided. |
| --- | --- |
| Sequence generation. | Quote: “patients were randomly allocated”.  Comment: Probably done, since earlier reports from the same investigators clearly describe use of random sequences (Cartwright 1980). |
| Sequence generation. | Quote: “patients were randomly allocated”.  Comment: Probably not done, as a similar trial by these investigators included the same phrase yet used alternate allocation (Winrow 1983). |
| Sequence generation. | Quote (from report): “patients were randomly allocated”.  Quote (from correspondence): “Randomization was performed according to day of treatment”.  Comment: Not randomized |

The judgement

Review authors’ judgements involve answering a specific question for each entry. In all cases, an answer ‘Yes’ indicates a low risk of bias, and an answer ‘No’ indicates high risk of bias.

If insufficient detail is reported of what happened in the study, the judgement will usually be ‘Unclear’ risk of bias. An ‘Unclear’ judgement should also be made if what happened in the study is known, but the risk of bias is unknown.

Criteria for judging risk of bias in the ‘Risk of bias’ assessment tool

| SEQUENCE GENERATION  Was the allocation sequence adequately generated? [Short form: Adequate sequence generation?] | |
| --- | --- |
| Criteria for a judgement of ‘YES’ (i.e. low risk of bias). | The investigators describe a random component in the sequence generation process such as:   - Referring to a random number table; - Using a computer random number generator; - Coin tossing; - Shuffling cards or envelopes; - Throwing dice; - Drawing of lots; - Minimization*.      *Minimization may be implemented without a random element, and this is considered to be equivalent to being random. |
| Criteria for the judgement of ‘NO’ (i.e. high risk of bias). | The investigators describe a non-random component in the sequence generation process. Usually, the description would involve some systematic, non-random approach, for example:   - Sequence generated by odd or even date of birth; - Sequence generated by some rule based on date (or day) of admission; - Sequence generated by some rule based on hospital or clinic record number.     Other non-random approaches happen much less frequently than the systematic approaches mentioned above and tend to be obvious.  They usually involve judgement or some method of non-random categorization of participants, for example:   - Allocation by judgement of the clinician; - Allocation by preference of the participant; - Allocation based on the results of a laboratory test or a series of tests; - Allocation by availability of the intervention. |
| Criteria for the judgement of ‘UNCLEAR’ (uncertain risk of bias). | Insufficient information about the sequence generation process to permit judgement of ‘Yes’ or ‘No’. |
| ALLOCATION CONCEALMENT  Was allocation adequately concealed? [Short form: Allocation concealment?] | |
| Criteria for a judgement of ‘YES’ (i.e. low risk of bias). | Participants and investigators enrolling participants could not foresee assignment because one of the following, or an equivalent method, was used to conceal allocation:   - Central allocation (including telephone, web-based, and pharmacy-controlled, randomization); - Sequentially numbered drug containers of identical appearance; - Sequentially numbered, opaque, sealed envelopes. |
| Criteria for the judgement of ‘NO’ (i.e. high risk of bias). | Participants or investigators enrolling participants could possibly foresee assignments and thus introduce selection bias, such as allocation based on:   - Using an open random allocation schedule (e.g. a list of random numbers); - Assignment envelopes were used without appropriate safeguards (e.g. if envelopes were unsealed or non­opaque or not sequentially numbered); - Alternation or rotation; - Date of birth; - Case record number; - Any other explicitly unconcealed procedure. |
| Criteria for the judgement of ‘UNCLEAR’ (uncertain risk of bias). | Insufficient information to permit judgement of ‘Yes’ or ‘No’. This is usually the case if the method of concealment is not described or not described in sufficient detail to allow a definite judgement – for example if the use of assignment envelopes is described, but it remains unclear whether envelopes were sequentially numbered, opaque and sealed. |
| BLINDING OF PARTICIPANTS, PERSONNEL AND OUTCOME ASSESSORS  Was knowledge of the allocated interventions adequately prevented during the study? [Short form: Blinding?] | |
| Criteria for a judgement of ‘YES’ (i.e. low risk of bias). | Any one of the following:   - No blinding, but the review authors judge that the outcome and the outcome measurement are not likely to be influenced by lack of blinding; - Blinding of participants and key study personnel ensured, and unlikely that the blinding could have been broken; - Either participants or some key study personnel were not blinded, but outcome assessment was blinded and the non-blinding of others unlikely to introduce bias. |
| Criteria for the judgement of ‘NO’ (i.e. high risk of bias). | Any one of the following:   - No blinding or incomplete blinding, and the outcome or outcome measurement is likely to be influenced by lack of blinding; - Blinding of key study participants and personnel attempted, but likely that the blinding could have been broken; - Either participants or some key study personnel were not blinded, and the non-blinding of others likely to introduce bias. |
| Criteria for the judgement of ‘UNCLEAR’ (uncertain risk of bias). | Any one of the following:   - Insufficient information to permit judgement of ‘Yes’ or ‘No’; - The study did not address this outcome. |
| INCOMPLETE OUTCOME DATA  Were incomplete outcome data adequately addressed? [Short form: Incomplete outcome data addressed?] | |
| Criteria for a judgement of ‘YES’ (i.e. low risk of bias). | Any one of the following:   - No missing outcome data; - Reasons for missing outcome data unlikely to be related to true outcome (for survival data, censoring unlikely to be introducing bias); - Missing outcome data balanced in numbers across intervention groups, with similar reasons for missing data across groups; - For dichotomous outcome data, the proportion of missing outcomes compared with observed event risk not enough to have a clinically relevant impact on the intervention effect estimate; - For continuous outcome data, plausible effect size (difference in means or standardized difference in means) among missing outcomes not enough to have a clinically relevant impact on observed effect size; - Missing data have been imputed using appropriate methods. |
| Criteria for the judgement of ‘NO’ (i.e. high risk of bias). | Any one of the following:   - Reason for missing outcome data likely to be related to true outcome, with either imbalance in numbers or reasons for missing data across intervention groups; - For dichotomous outcome data, the proportion of missing outcomes compared with observed event risk enough to induce clinically relevant bias in intervention effect estimate; - For continuous outcome data, plausible effect size (difference in means or standardized difference in means) among missing outcomes enough to induce clinically relevant bias in observed effect size; - ‘As-treated’ analysis done with substantial departure of the intervention received from that assigned at randomization; - Potentially inappropriate application of simple imputation. |
| Criteria for the judgement of ‘UNCLEAR’ (uncertain risk of bias). | Any one of the following:   - Insufficient reporting of attrition/exclusions to permit judgement of ‘Yes’ or ‘No’ (e.g. number randomized not stated, no reasons for missing data provided); - The study did not address this outcome. |
| SELECTIVE OUTCOME REPORTING  Are reports of the study free of suggestion of selective outcome reporting? [Short form: Free of selective reporting?] | |
| Criteria for a judgement of ‘YES’ (i.e. low risk of bias). | Any of the following:   - The study protocol is available and all of the study’s pre-specified (primary and secondary) outcomes that are of interest in the review have been reported in the pre-specified way; - The study protocol is not available but it is clear that the published reports include all expected outcomes, including those that were pre-specified (convincing text of this nature may be uncommon). |
| Criteria for the judgement of ‘NO’ (i.e. high risk of bias). | Any one of the following:   - Not all of the study’s pre-specified primary outcomes have been reported; - One or more primary outcomes is reported using measurements, analysis methods or subsets of the data (e.g. subscales) that were not pre-specified; - One or more reported primary outcomes were not pre-specified (unless clear justification for their reporting is provided, such as an unexpected adverse effect); - One or more outcomes of interest in the review are reported incompletely so that they cannot be entered in a meta-analysis; - The study report fails to include results for a key outcome that would be expected to have been reported for such a study. |
| Criteria for the judgement of ‘UNCLEAR’ (uncertain risk of bias). | Insufficient information to permit judgement of ‘Yes’ or ‘No’. It is likely that the majority of studies will fall into this category. |
| OTHER POTENTIAL THREATS TO VALIDITY  Was the study apparently free of other problems that could put it at a risk of bias? [Short form: Free of other bias?] | |
| Criteria for a judgement of ‘YES’ (i.e. low risk of bias). | The study appears to be free of other sources of bias. |
| Criteria for the judgement of ‘NO’ (i.e. high risk of bias). | There is at least one important risk of bias. For example, the study:   - Had a potential source of bias related to the specific study design used; or - Stopped early due to some data-dependent process (including a formal-stopping rule); or - Had extreme baseline imbalance; or - Has been claimed to have been fraudulent; or - Had some other problem. |
| Criteria for the judgement of ‘UNCLEAR’ (uncertain risk of bias). | There may be a risk of bias, but there is either:   - Insufficient information to assess whether an important risk of bias exists; or - Insufficient rationale or evidence that an identified problem will introduce bias. |
