# Supplementary material for: Effects of whole-body vibration on postural control in elderly: a systematic review and meta-analysis
Source: BMC Geriatr. 2011 Nov 3;11:72. doi: 10.1186/1471-2318-11-72 (PMC3229447; doi:10.1186/1471-2318-11-72)
Supplement: Additional file 2 — Forest plot of 3 trials comparing the effects of any type of vibration and control interventions on mixed balance. The analyses were separated for trials reporting post-values (i.e. mean and SD from follow-up) and for trials that reported change values (i.e. mean and SD from the changes from baseline to follow-up). Random effects model with predictive interval. The predictive interval indicates the range within which we expect the effects of 95% of future studies. Values on x-axis denote SMDs. [file 1471-2318-11-72-S2.PPT]

## Slide 1
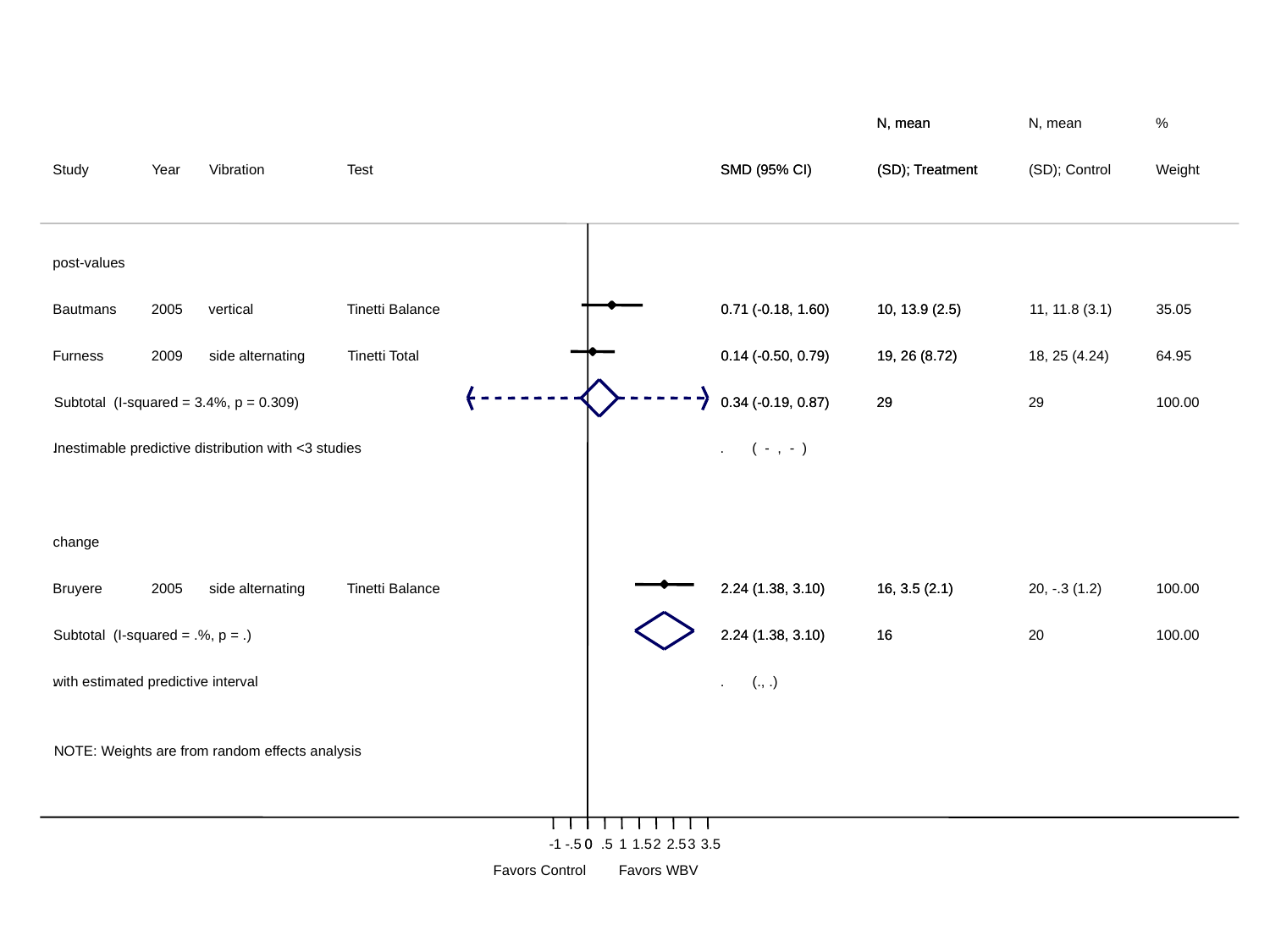

N, mean
N, mean
N, mean
%
Study
Year
Vibration
Test
SMD (95% CI)
SMD (95% CI)
(SD); Treatment
(SD); Treatment
(SD); Control
Weight
post-values
Bautmans
2005
vertical
Tinetti Balance
0.71 (-0.18, 1.60)
0.71 (-0.18, 1.60)
10, 13.9 (2.5)
10, 13.9 (2.5)
11, 11.8 (3.1)
35.05
Furness
2009
side alternating
Tinetti Total
0.14 (-0.50, 0.79)
0.14 (-0.50, 0.79)
19, 26 (8.72)
19, 26 (8.72)
18, 25 (4.24)
64.95
Subtotal (I-squared = 3.4%, p = 0.309)
0.34 (-0.19, 0.87)
0.34 (-0.19, 0.87)
29
29
29
100.00
.
Inestimable predictive distribution with <3 studies
. ( - , - )
change
Bruyere
2005
side alternating
Tinetti Balance
2.24 (1.38, 3.10)
2.24 (1.38, 3.10)
16, 3.5 (2.1)
16, 3.5 (2.1)
20, -.3 (1.2)
100.00
Subtotal (I-squared = .%, p = .)
2.24 (1.38, 3.10)
2.24 (1.38, 3.10)
16
16
20
100.00
.
with estimated predictive interval
. (., .)
NOTE: Weights are from random effects analysis
-1
-.5
0
0
.5
1
1.5
2
2.5
3
3.5
Favors Control
Favors WBV
