# Supplementary material for: Effects of whole-body vibration on postural control in elderly: a systematic review and meta-analysis
Source: BMC Geriatr. 2011 Nov 3;11:72. doi: 10.1186/1471-2318-11-72 (PMC3229447; doi:10.1186/1471-2318-11-72)
Supplement: Additional file 3 — Forest plot of 11 trials (12 comparisons comparing) stratified for the vibration type (vertical and side alternating). Outcomes were tests for dynamic balance. The analyses were separated for trials reporting post-values (i.e. mean and SD from follow-up) and for trials that reported change values (i.e. mean and SD from the changes from baseline to follow-up). Random effects model with predictive interval. The predictive interval indicates the range within which we expect the effects of 95% of future studies will be. Values on x-axis denote SMDs. [file 1471-2318-11-72-S3.PPT]

## Slide 1
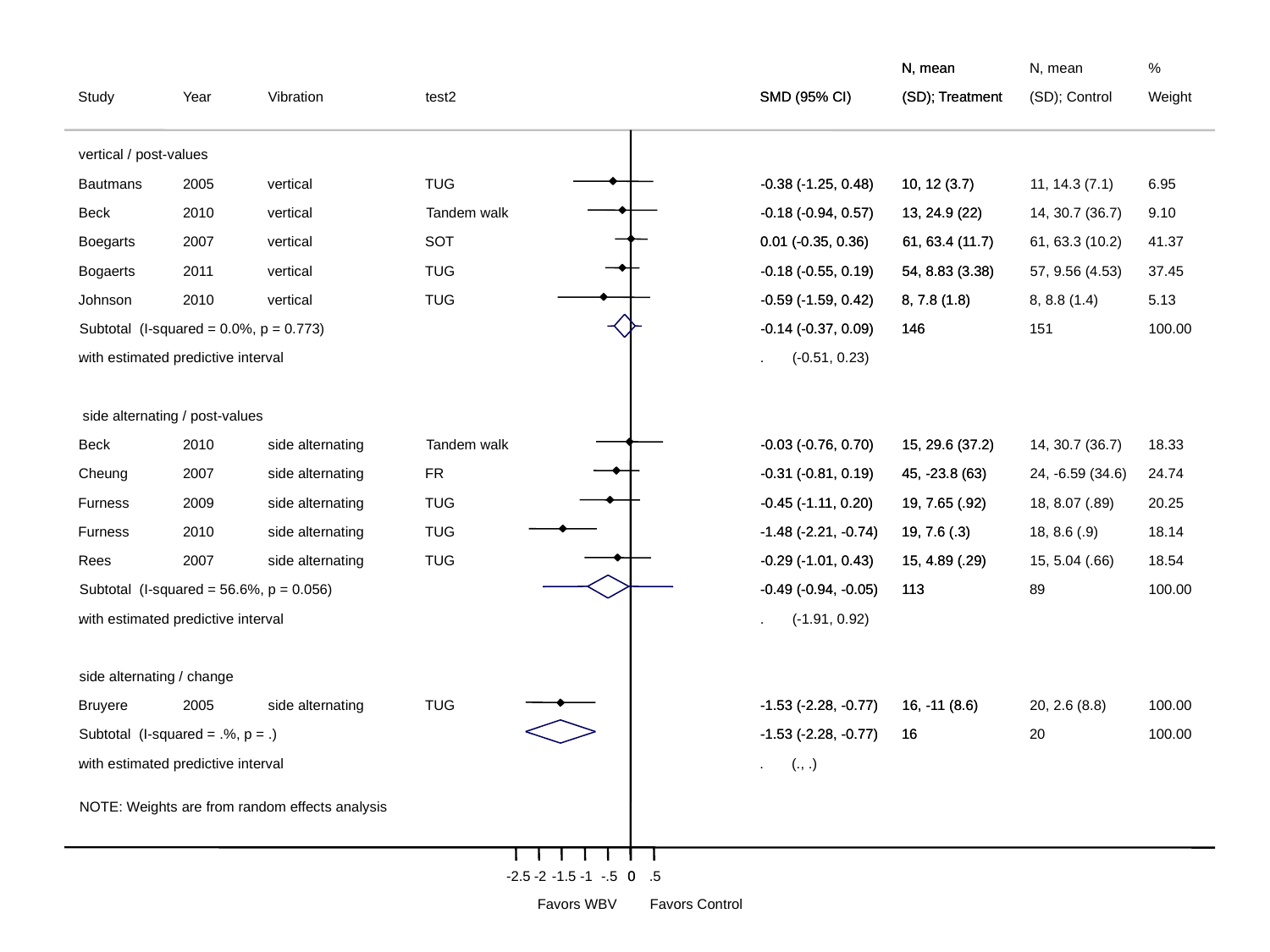

N, mean
N, mean
N, mean
%
Study
Year
Vibration
test2
SMD (95% CI)
SMD (95% CI)
(SD); Treatment
(SD); Treatment
(SD); Control
Weight
vertical / post-values
Bautmans
2005
vertical
TUG
-0.38 (-1.25, 0.48)
-0.38 (-1.25, 0.48)
10, 12 (3.7)
10, 12 (3.7)
11, 14.3 (7.1)
6.95
Beck
2010
vertical
Tandem walk
-0.18 (-0.94, 0.57)
-0.18 (-0.94, 0.57)
13, 24.9 (22)
13, 24.9 (22)
14, 30.7 (36.7)
9.10
Boegarts
2007
vertical
SOT
0.01 (-0.35, 0.36)
0.01 (-0.35, 0.36)
61, 63.4 (11.7)
61, 63.4 (11.7)
61, 63.3 (10.2)
41.37
Bogaerts
2011
vertical
TUG
-0.18 (-0.55, 0.19)
-0.18 (-0.55, 0.19)
54, 8.83 (3.38)
54, 8.83 (3.38)
57, 9.56 (4.53)
37.45
Johnson
2010
vertical
TUG
-0.59 (-1.59, 0.42)
-0.59 (-1.59, 0.42)
8, 7.8 (1.8)
8, 7.8 (1.8)
8, 8.8 (1.4)
5.13
Subtotal (I-squared = 0.0%, p = 0.773)
-0.14 (-0.37, 0.09)
-0.14 (-0.37, 0.09)
146
146
151
100.00
.
with estimated predictive interval
. (-0.51, 0.23)
side alternating / post-values
Beck
2010
side alternating
Tandem walk
-0.03 (-0.76, 0.70)
-0.03 (-0.76, 0.70)
15, 29.6 (37.2)
15, 29.6 (37.2)
14, 30.7 (36.7)
18.33
Cheung
2007
side alternating
FR
-0.31 (-0.81, 0.19)
-0.31 (-0.81, 0.19)
45, -23.8 (63)
45, -23.8 (63)
24, -6.59 (34.6)
24.74
Furness
2009
side alternating
TUG
-0.45 (-1.11, 0.20)
-0.45 (-1.11, 0.20)
19, 7.65 (.92)
19, 7.65 (.92)
18, 8.07 (.89)
20.25
Furness
2010
side alternating
TUG
-1.48 (-2.21, -0.74)
-1.48 (-2.21, -0.74)
19, 7.6 (.3)
19, 7.6 (.3)
18, 8.6 (.9)
18.14
Rees
2007
side alternating
TUG
-0.29 (-1.01, 0.43)
-0.29 (-1.01, 0.43)
15, 4.89 (.29)
15, 4.89 (.29)
15, 5.04 (.66)
18.54
Subtotal (I-squared = 56.6%, p = 0.056)
-0.49 (-0.94, -0.05)
-0.49 (-0.94, -0.05)
113
113
89
100.00
.
with estimated predictive interval
. (-1.91, 0.92)
side alternating / change
Bruyere
2005
side alternating
TUG
-1.53 (-2.28, -0.77)
-1.53 (-2.28, -0.77)
16, -11 (8.6)
16, -11 (8.6)
20, 2.6 (8.8)
100.00
Subtotal (I-squared = .%, p = .)
-1.53 (-2.28, -0.77)
-1.53 (-2.28, -0.77)
16
16
20
100.00
.
with estimated predictive interval
. (., .)
NOTE: Weights are from random effects analysis
-2.5
-2
-1.5
-1
-.5
0
0
.5
Favors WBV
Favors Control
